# Assembly and Comparative Analysis of Five Complete Mitochondrial Genomes of the *Aquilegia ecalcarata* Complex

**DOI:** 10.3390/ijms27156589

**Published:** 2026-07-24

**Authors:** Yao Xiao, Fang Liu, Guixia Xu, Xi He

**Affiliations:** 1 State Key Laboratory for Development and Utilization of Forest Food Resources, Zhejiang A&F University, Hangzhou 311300, China; xiaoyao@ibcas.ac.cn (Y.X.); liufang@ibcas.ac.cn (F.L.); 2 Zhejiang Key Laboratory of Non-Wood Forest Products Quality Regulation and Processing Utilization, Zhejiang A&F University, Hangzhou 311300, China; 3 State Key Laboratory of Plant Diversity and Specialty Crops, Institute of Botany, Chinese Academy of Sciences, Beijing 100093, China; 4 Key Laboratory of Systematic and Evolutionary Botany, Institute of Botany, Chinese Academy of Sciences, Beijing 100093, China

**Keywords:** *Aquilegia ecalcarata* complex, mitochondrial genome, parallel evolution, spurless phenotype

## Abstract

The *Aquilegia ecalcarata* (*A. ecalcarata*) complex is a valuable evolutionary model for studying parallel evolution, introgression, and incomplete lineage sorting. Deciphering this complexity requires evidence from multiple genomic compartments. While substantial nuclear and chloroplast resources exist, mitochondrial genome data, which capture the maternally inherited evolutionary history, have been absent for this group. Using long-read sequencing data, this study assembled and comprehensively characterized the mitochondrial genomes of five representative taxa within the *A. ecalcarata* complex. Comparative analyses reveal that the two spurless lineages, *A. ecalcarata* clade A and clade B, do not form a monophyletic group based on mitochondrial phylogeny. Instead, each clusters with a different spurred relative, consistent with the independent evolution of spur loss. This finding is further supported by collinearity patterns, gene content, and lineage-specific MTPT distributions. By providing the first mitochondrial genomic resource for this group, this study offers independent organellar evidence for the multiple origins of spurless phenotype in the *A. ecalcarata* complex.

## 1. Introduction

Mitochondria are indispensable organelles in eukaryotic cells, playing pivotal roles in energy metabolism, programmed cell death, and cellular signal transduction [1,2,3,4]. Mitochondria harbor their own independent genomes. The coding regions of the mitochondrial genome (mtDNA) encode subunits of enzymes that play crucial roles in the respiratory electron transport chain, as well as components necessary for mitochondrial translation machinery [5,6]. In contrast to animals and fungi, plant mitochondrial genomes display remarkable distinctiveness in size, structure, and evolutionary processes. Plant mtDNA varies extensively in size, ranging from 66 kb (*Viscum scurruloideum*) to 19 Mb (*Cathaya argyrophylla*) [7,8]. The predominant portion of the plant mitochondrial genome comprises non-coding sequences, which serve as the primary drivers of size variation. The abundance of diverse repetitive sequences of varying lengths renders plant mtDNA highly prone to recombination, leading to frequent structural rearrangements [5,9,10]. Additionally, horizontal gene transfer and intracellular gene transfer further augment the sequence and structural diversity of plant mtDNA [11,12,13]. Among these, a prominent form of intracellular gene transfer is the movement of DNA from chloroplasts to mitochondria, generating fragments known as mitochondrial-plastid DNAs (MTPTs) [13]. Despite the high variability of non-coding sequences, the genes in plant mtDNA are highly conserved, with mutation rates lower than those of nuclear and chloroplast genes [5,14,15]. The diverse non-coding regions and conserved genes make mtDNA a valuable molecular resource for investigating evolutionary relationships across species.

The genus *Aquilegia* (Ranunculaceae) is widely distributed across temperate regions of the Northern Hemisphere and comprises at least 70 species. Several biological characteristics render *Aquilegia* a common model system in evolutionary biology [16,17,18]. *Aquilegia* represents an early-diverging eudicot lineage that originated close to the eudicot–monocot split (~120 Mya), thus occupying a pivotal phylogenetic position between monocots and core eudicots [17]. The genus has undergone two independent rapid adaptive radiations, resulting in its widespread distribution [19,20]. Reproductive isolation is often incomplete among species [21,22], which facilitates frequent interspecific hybridization and introgression. Critically, *Aquilegia* exhibits distinctive floral organ morphology, including petaloid sepals, staminodium, and petals with nectar spurs. The nectar spur, a key innovative trait, is considered a major driver of these radiations. In North American taxa, spur-length diversification correlates with pollinator shifts, promoting reproductive isolation and accelerating speciation [23,24].

Phylogenomic reconstructions indicate that the genus originated in East Asia approximately 6.9 million years ago, and following the split of two major clades, one lineage colonized North America while the other diversified across Eurasia [20]. Interestingly, the Eurasian lineage that later re-migrated back into East Asia gave rise to a group of species that challenge the classical narrative of spur-driven diversification, most notably the *Aquilegia ecalcarata* (*A*. *ecalcarata*) complex. *A*. *ecalcarata*, endemic to China, is traditionally recognized as the only species within the genus that completely lacks nectar spurs [25]. However, multiple molecular phylogenetic studies have revealed that this morphologically defined “species” is not monophyletic. Populations exhibiting the spurless trait are instead recovered in multiple distinct clades, providing strong evidence that the loss of nectar spurs has occurred independently on at least two occasions [26,27,28,29,30,31]. A recent phylogenomic study based on genome-wide resequencing data further resolved the complex into two major clades, each comprising a mixture of spurred *Aquilegia kansuensis* (*A. kansuensis*) and spurless *A. ecalcarata* individuals, alongside spurred taxa such as *Aquilegia yangii* (*A. yangii*), *Aquilegia yangii* (*A. yabeana*), and *Aquilegia rockii* (*A. rockii*) [31]. This parallel loss of a key innovative trait stands in striking contrast to the adaptive elongation of spurs observed in North American *Aquilegia*. Although nuclear and chloroplast genome resources have contributed substantially to our understanding of this complex, mitochondrial genome data remain extremely limited for the genus. To date, only the mitochondrial genome of *A. amurensis* has been published, and no mitogenomic resources are available for the *A*. *ecalcarata* complex. Here, we used third-generation sequencing data to assemble and comprehensively characterize the mitochondrial genomes of five taxa representing both clades and both the spurless and spurred morphologies: *A. kansuensis* and *A. ecalcarata* from clade A, *A. ecalcarata* from clade B, *A. yangii*, and *A. yabeana*. Our study aims to provide the first mitochondrial genetic resource for the *A. ecalcarata* complex, filling a critical gap for future organellar-level phylogenetic and evolutionary investigations of this group.

## 2. Results

### 2.1. Mitochondrial Genome Assembly and Gene Annotation

**Figure 1:**
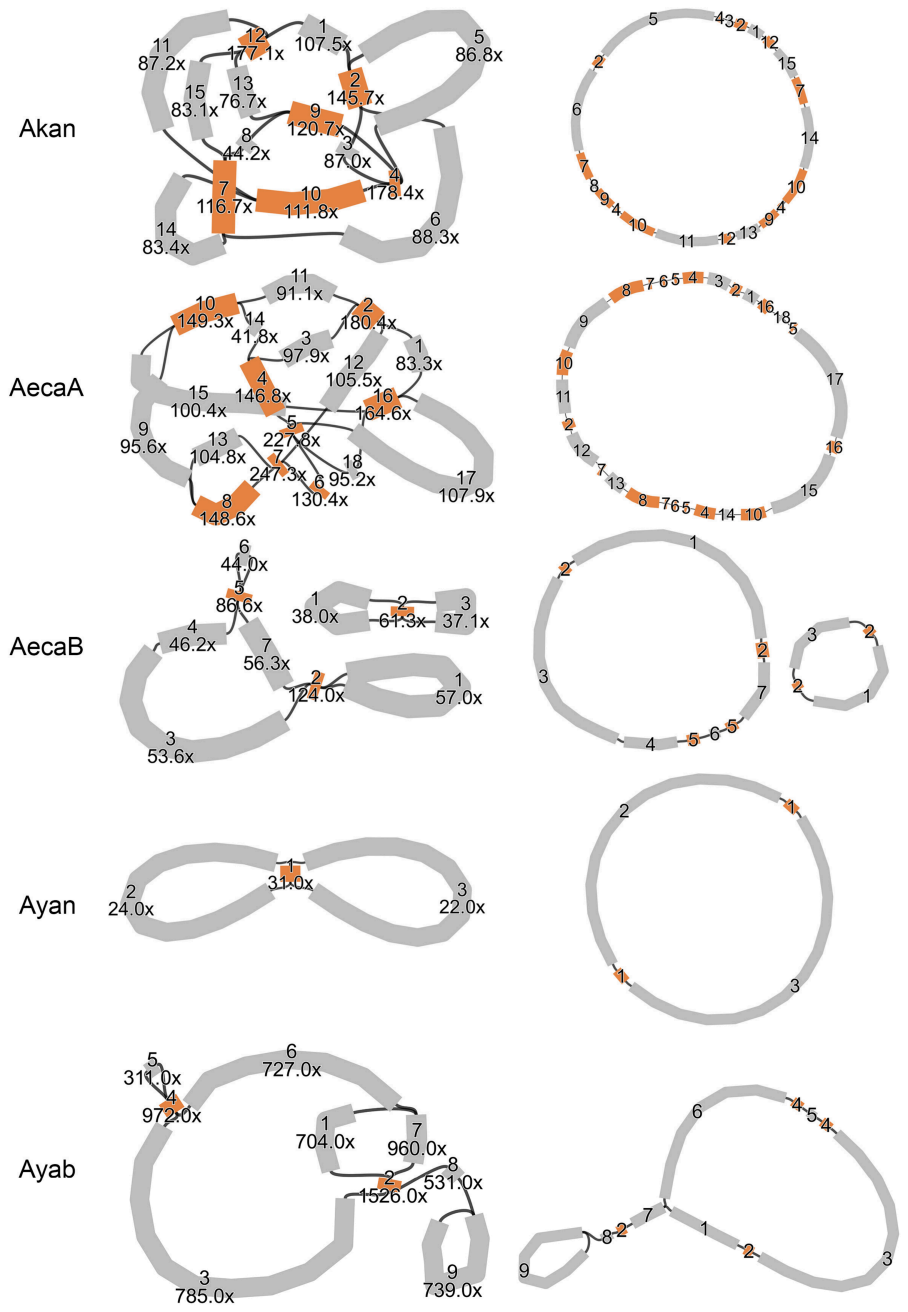
Schematic of the mitochondrial genome contigs for *A. kansuensis* (Akan), *A. ecalcarata* clade A (AecaA), *A. ecalcarata* clade B (AecaB), *A. yangii* (Ayan), and *A. yabeana* (Ayab). Raw assembly graphs (**left** panel) show contig connectivity and depth (as output by the respective assemblers) for each genome. Representative structures (**right** panel) derived from the assembly graphs based on contig connectivity and depth. Contig-ID and contig depth are annotated as digit in raw assembly graphs (**left** panel). Contig-ID are annotated as digit in representative structures (**right** panel). Orange indicates contigs that are duplicated in both raw assembly graphs and representative structures.

Using PacBio-HiFi data (Table S1), we successfully assembled the mitochondrial genomes of *A. kansuensis*, *A. ecalcarata* clade A, *A. ecalcarata* clade B, and *A. yabeana* within the *A*. *ecalcarata* complex. The mitochondrial genome of *A. yangii* was assembled using published PacBio long-read data (NCBI BioProject: PRJNA636213, Table S1). De novo assembly yielded 15, 18, 10, 4, and 9 contigs for *A. kansuensis*, *A. ecalcarata* clade A, *A. ecalcarata* clade B, *A. yangii*, and *A. yabeana*, respectively, revealing complex genome architectures (Figure 1). To facilitate downstream analyses, we joined the contigs into longer representative sequences based on contig connectivity and read depth (Figure 1 and Figure S1), with all inter-contig junctions supported by long spanning reads (Figure S1, Table S2). The mitochondrial genomes of *A. kansuensis*, *A. ecalcarata* clade A, and *A. yangii* could each be organized as a single circular molecule (Figure 1 and Figure S1), with total lengths of 618,203 bp (GC content 46.31%), 618,154 bp (GC content 46.31%), and 488,419 bp (GC content 46.31%), respectively (Table 1). The contigs of the mitochondrial genome of *A. ecalcarata* clade B could be grouped as two unconnected sets, and no long reads with appropriate depth could bridge the gap between them. Thus, the mitochondrial genome of *A. ecalcarata* clade B was resolved as two distinct circular molecules (Figure 1): a larger ring of 420,695 bp (GC content 45.81%, depth 54.5×) and a small ring of 138,623 bp (GC content 46.14%, depth 37.4×), totaling 559,318 bp (GC content 45.98%) (Table 1). For *A. yabeana*, the contig connectivity and read depth information did not support a circular conformation, but rather indicated a complex molecular structure (Figure 1). To obtain a continuous reference sequence for downstream analyses and to present the genome clearly, we selected the most strongly supported connections, omitting the links between contig1 and contig7, and resolved the ambiguous internal connections within contig9 by treating them as breakpoints (Figure 1 and Figure S1). The resulting representation is a linear molecule of 568,238 bp with a GC content of 45.99% (Table 1). We note, however, that the in vivo structure may be more complex.

**Figure 2:**
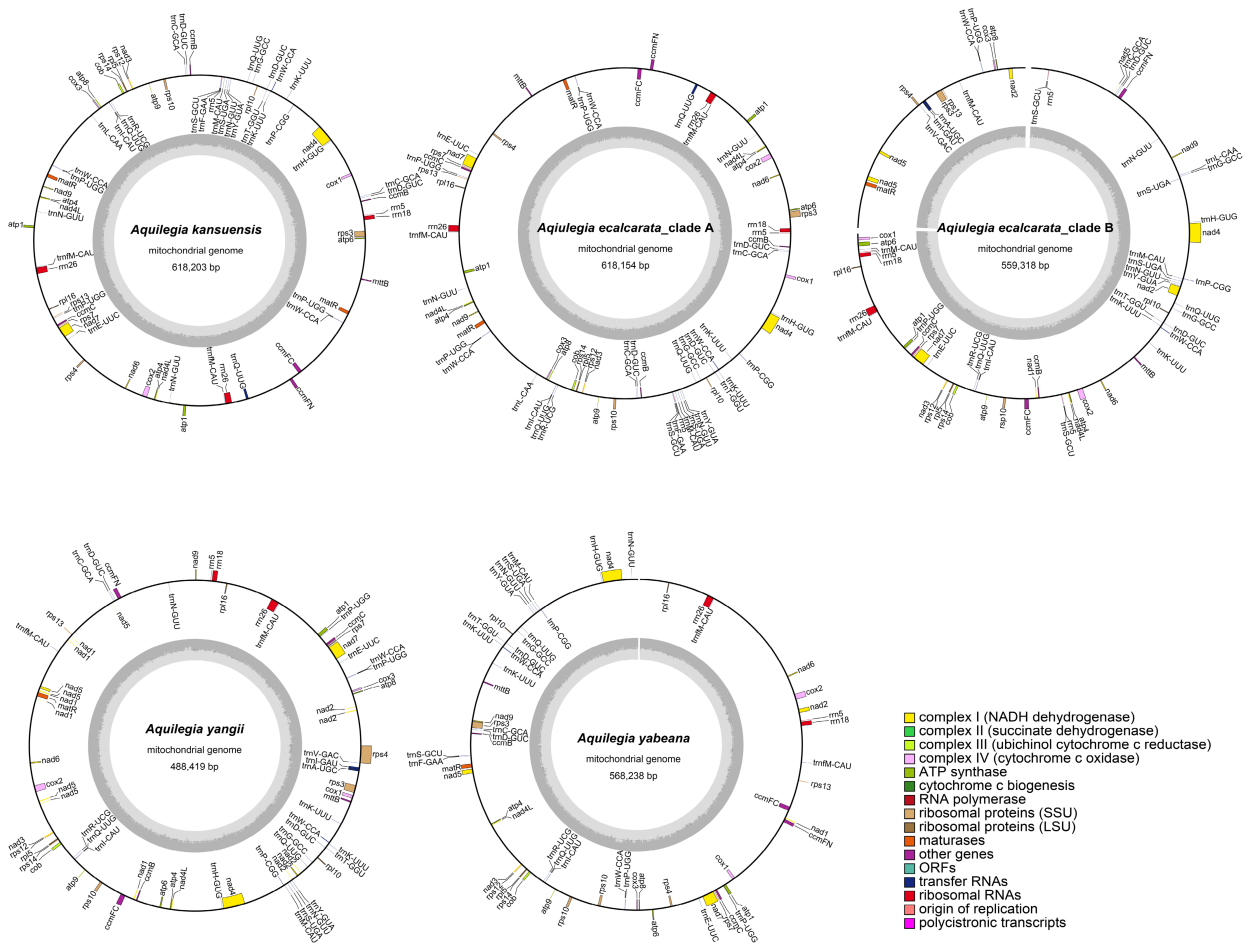
Schematics of the mitochondrial genomes of *A. kansuensis*, *A. ecalcarata* clade A, *A. ecalcarata* clade B, *A. yangii,* and *A. yabeana*. Genes marked outside rings are on the direct strand, whereas the genes inside rings are on the reverse strand. Genes are color-coded according to their functional categories.

Across the five mitochondrial genomes, we identified 35 distinct protein-coding genes (PCGs), comprising 24 core PCGs and 11 non-core PCGs (Table S3, Figure 2 and Figures S2–S6). Copy numbers of several PCGs varied among taxa. *A. kansuensis* and *A. ecalcarata* clade A both contained two copies of *atp1*, *atp4*, *nad4L*, *ccmB*, and *matR*, whereas these genes were present as single copies in *A. ecalcarata* clade B, *A. yangii*, and *A. yabeana* (Table S3). The *rps10* gene was duplicated in *A. yabeana* but was a single copy in the other four taxa (Table S3). The five mitochondrial genomes harbored 21, 21, 23, 21, and 20 unique tRNA genes, respectively. *A. kansuensis* and *A. ecalcarata* clade A exhibited identical tRNA gene sets and copy numbers (Table S3). Three unique rRNA genes (*rrn5*, *rrn18*, and *rrn26*) were identified in all five mitochondrial genomes, although copy numbers differed slightly among them. The *sdh4* gene was identified as a pseudogene in all five mitochondrial genomes (Table S3), each characterized by an internal premature stop codon. The predicted coding sequence (CDS) of *sdh4* is 375 bp in *A. yabeana* and 381 bp in the other four species. However, all five taxa share an in-frame premature termination codon (TAA) at nucleotide positions 175–177, which could cause translation to terminate at codon 59, resulting in a severely truncated protein of only 58 amino acids. Comparison with closely related species, including *Paropyrum anemonoides* (*P. anemonoides*) and *Coptis chinensis* (*C. chinensis*), showed that the corresponding codon is TAC (tyrosine), indicating that the TAA stop codon in the *A. ecalcarata* complex arose from a C-to-A point mutation rather than from an RNA editing site. Since plant mitochondrial RNA editing is almost exclusively of the C-to-U type, this premature stop codon cannot be restored to a sense codon by editing.

### 2.2. Analysis of Repeat Sequences

**Figure 3:**
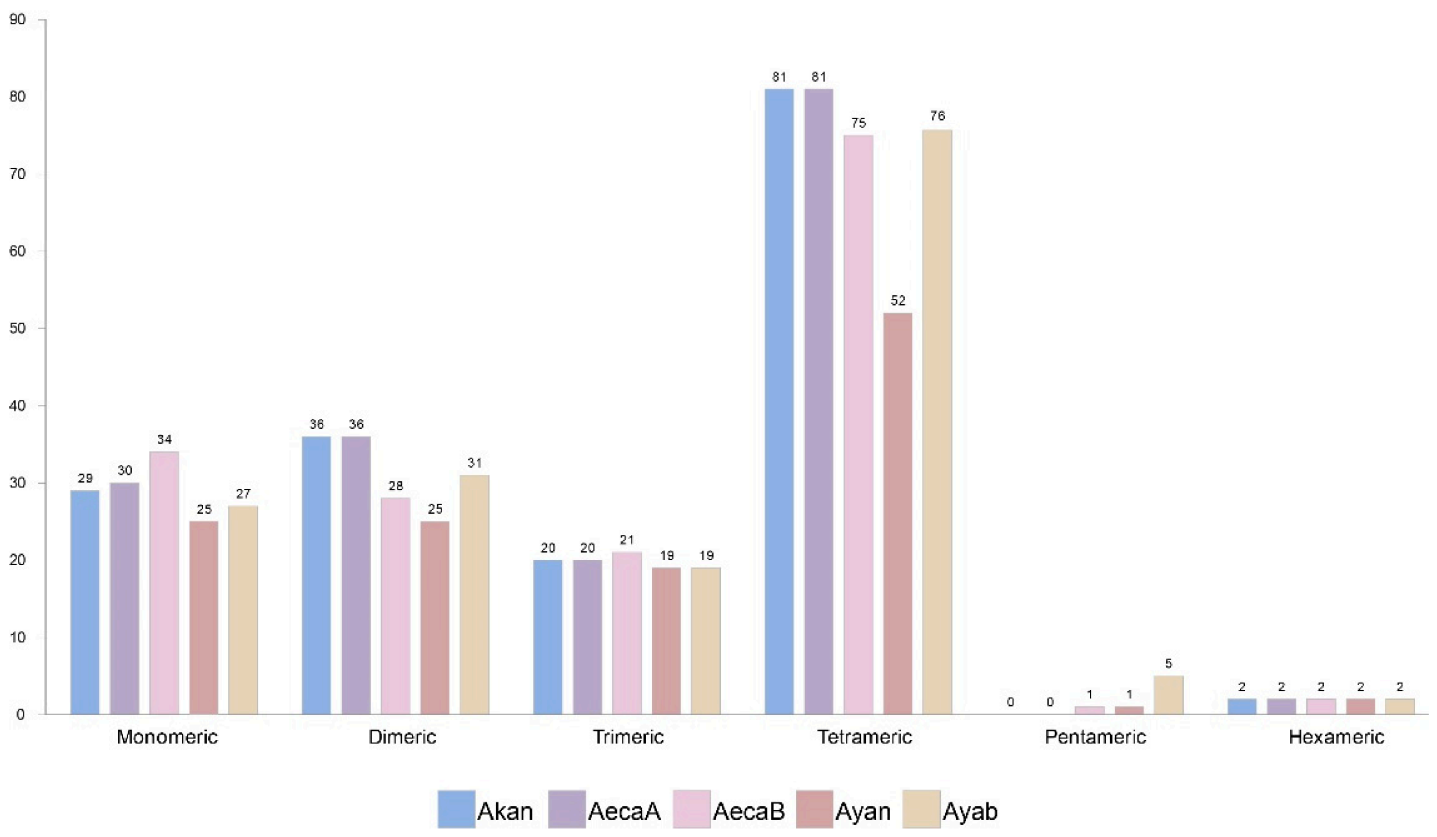
Types and numbers of simple sequence repeats (SSRs) in the mitochondrial genomes of *A. kansuensis* (Akan), *A. ecalcarata* clade A (AecaA), *A. ecalcarata* clade B (AecaB), *A. yangii* (Ayan), and *A. yabeana* (Ayab).

Simple sequence repeat (SSR) analysis identified 168, 169, 161, 124, and 160 SSRs in *A. kansuensis*, *A. ecalcarata* clade A, *A. ecalcarata* clade B, *A. yangii*, and *A. yabeana*, respectively. The overall distribution of SSR types was broadly similar across the five taxa (Figure 3). Tetrameric SSRs constituted the most abundant type, accounting for approximately 50% of the total in each genome (Figure 3). Monomeric, dimeric, and trimeric SSRs were moderately abundant, with individual counts ranging from 19 to 36 across the five taxa, whereas pentameric and hexameric SSRs were extremely rare (Figure 3).

**Figure 4:**
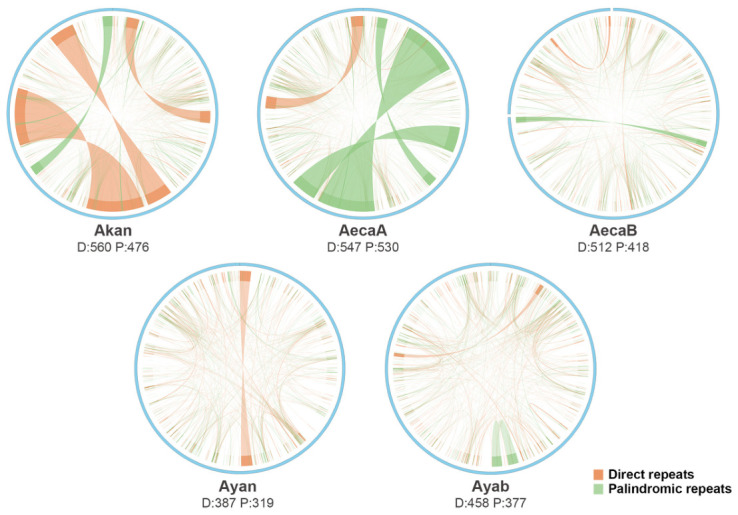
Distribution of dispersed repetitive sequences in the mitochondrial genomes of *A. kansuensis* (Akan), *A. ecalcarata* clade A (AecaA), *A. ecalcarata* clade B (AecaB), *A. yangii* (Ayan), and *A. yabeana* (Ayab). The numbers of direct (D, orange) and palindromic (P, green) repeats are shown below each circle.

The total numbers of dispersed repeat sequences are 1036, 1077, 930, 706, and 835 in *A. kansuensis*, *A. ecalcarata* clade A, *A. ecalcarata* clade B, *A. yangii*, and *A. yabeana*, respectively (Figure 4, Table S4). All detected dispersed repeats were either direct or palindromic, with both types occurring at similar frequencies. Approximately 90% of these repeats were shorter than 100 bp. Notably, all five mitogenomes harbored long (>1 kb) repeats (Table S5), which may mediate homologous recombination, potentially leading to the formation of alternative subgenomic conformations (Supplementary File S3).

### 2.3. Analysis of Chloroplast-Derived Regions

**Figure 5:**
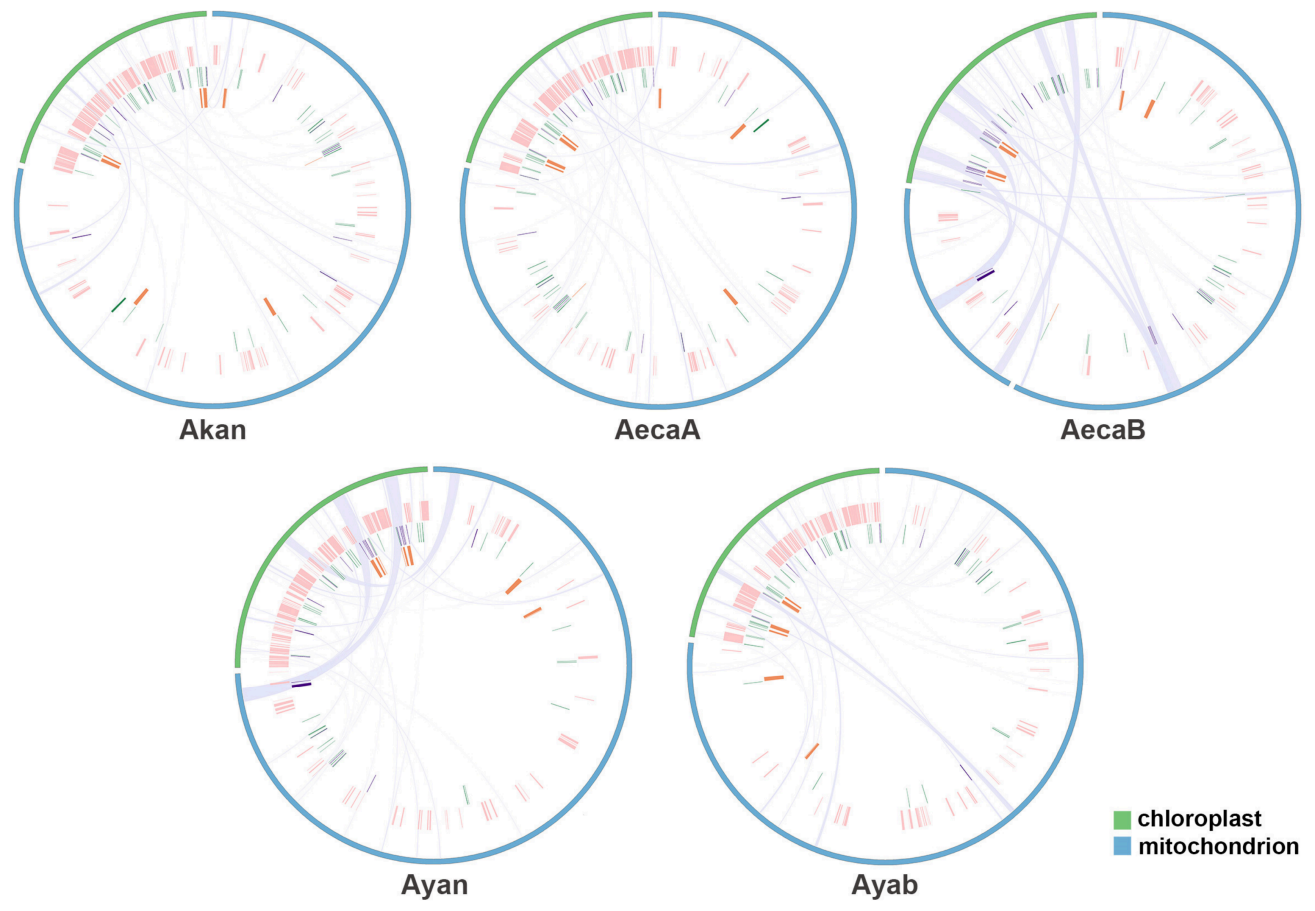
Synteny analysis between chloroplast and mitochondrial genomes in *A. kansuensis* (Akan), *A. ecalcarata* clade A (AecaA), *A. ecalcarata* clade B (AecaB), *A. yangii* (Ayan), and *A. yabeana* (Ayab). For each species, the circular structure is composed of green and blue segments, representing the chloroplast and mitochondrial genomes, respectively. Along the inner side of the circle, three concentric bands of colored bars indicate the positions of different gene categories: the pink, green, and orange bands represent PCGs, tRNA genes, and rRNA genes, respectively. MTPT regions are highlighted in purple.

Sequence comparison between each mitochondrial genome and its corresponding assembled and annotated chloroplast genome (Figure S7) identified a total of 20, 19, 22, 21, and 19 mitochondrial–plastid DNA (MTPTs) fragments in *A. kansuensis*, *A. ecalcarata* clade A, *A. ecalcarata* clade B, *A. yangii*, and *A. yabeana*, respectively (Figure 5, Table S6). MTPTs were identified using consistent criteria: an E-value < 1 × 10^−5^ and a sequence identity > 70%. The fragment lengths ranged from 43 to 2992 bp in *A. kansuensis*, *A. ecalcarata* clade A, and *A. yabeana*, whereas those in *A. ecalcarata* clade B and *A. yangii* extended up to 7572 bp and 6312 bp, respectively. Collectively, MTPTs accounted for 1.11%, 1.10%, 5.04%, 3.60%, and 1.47% of the corresponding mitochondrial genome sizes (Table S6). Annotation further revealed that the MTPTs of the five taxa contained 9, 9, 13, 9, and 7 unique tRNA genes, respectively, whereas no PCGs or rRNA genes were detected (Table S7, Figure S8). Among these, the tRNA genes *trnD-GUC*, *trnH-GUG*, *trnN-GUU*, *trnM-CAU, trnP-UGG* and *trnW-CCA* were present in the MTPTs of all five taxa (Figure S8). Notably, two copies of *trnP-UGG* were identified in the MTPTs of both *A. kansuensis* and *A. ecalcarata* clade A, and both copies were syntenic to the same chloroplast genomic region. The same duplication pattern was observed for *trnW-CCA* in these two taxa, as well as for *trnM-CAU* in *A. ecalcarata* clade B and *trnN-GUU* in *A. yabeana* (Figure S8). The presence of multiple MTPT copies that map to an identical chloroplast donor region suggests that, following the initial chloroplast-to-mitochondria DNA transfer, subsequent intramitochondrial duplication and translocation events have occurred.

### 2.4. Analysis of Codon Usage Bias

**Figure 6:**
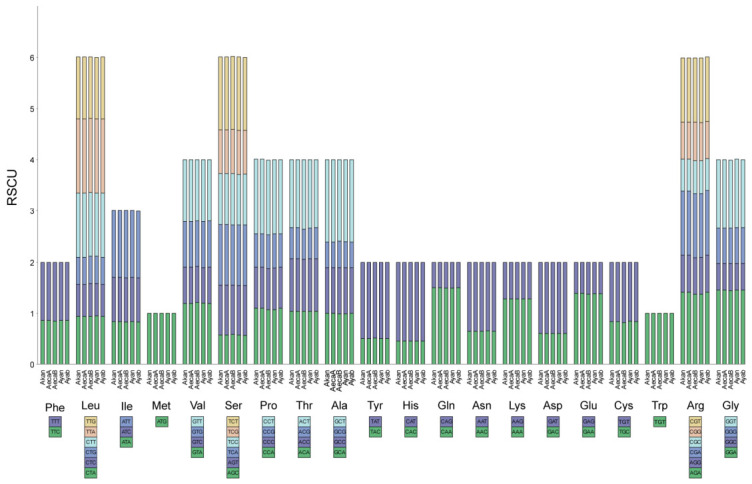
Relative synonymous codon usage (RSCU) values of the PCGs in mitochondrial genomes of *A. kansuensis* (Akan), *A. ecalcarata* clade A (AecaA), *A. ecalcarata* clade B (AecaB), *A. yangii* (Ayan), and *A. yabeana* (Ayab).

Codon usage bias was analyzed for the PCGs across the five *Aquilegia* mitogenomes. The overall codon usage patterns were highly conserved among the five taxa (Figure 6). Excluding methionine (Met) and tryptophan (Trp), which are each encoded by a single codon and thus uninformative for bias analysis, all other amino acids exhibited varying degrees of relative synonymous codon usage bias (RSCU). Alanine (Ala), glutamine (Gln), and histidine (His) displayed relatively strong codon bias, with maximum RSCU values of 1.61, 1.50, and 1.54, respectively (Table S8). In contrast, cysteine (Cys), phenylalanine (Phe), and valine (Val) exhibited weak codon bias, with maximum codon RSCU values of 1.16, 1.14, and 1.21, respectively (Table S8). Across all five taxa, high-frequency codons (RSCU > 1) preferentially used A or T at the third codon position, whereas low-frequency codons (RSCU < 1) were enriched for C or G at the third position (Table S8).

**Figure 7:**
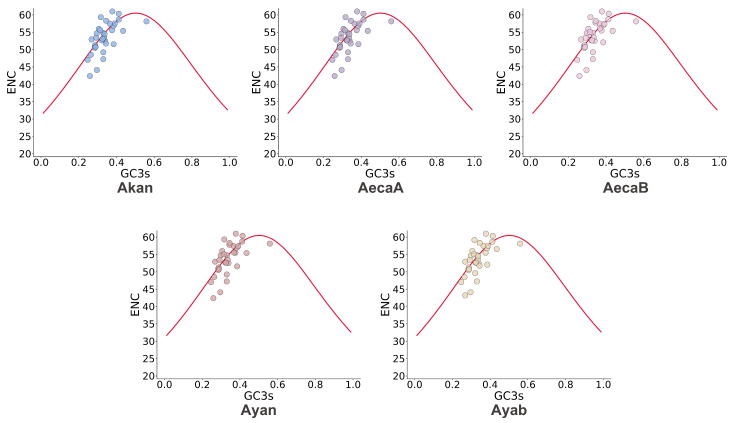
ENC plot of the PCGs in mitochondrial genomes of *A. kansuensis* (Akan), *A. ecalcarata* clade A (AecaA), *A. ecalcarata* clade B (AecaB), *A. yangii* (Ayan), and *A. yabeana* (Ayab). The red line is the expected ENC curve.

The analysis of effective number of codons (ENC) plotted against GC content at the third codon position (GC3s) (Figure 7) revealed that most genes fell close to or slightly above the expected curve, suggesting that GC3s-mediated mutational pressure plays a primary role in shaping codon usage. Notably, a subset of genes (7/34) deviated substantially below this curve, with an ENC deviation ratio exceeding 0.05 (Table S9), implying potential selective constraints acting on these genes. Collectively, these results are consistent with a combination of mutational bias and possible weak natural selection shaping codon usage across the five mitogenomes.

### 2.5. Prediction of RNA Editing Sites of PCGs

**Figure 8:**
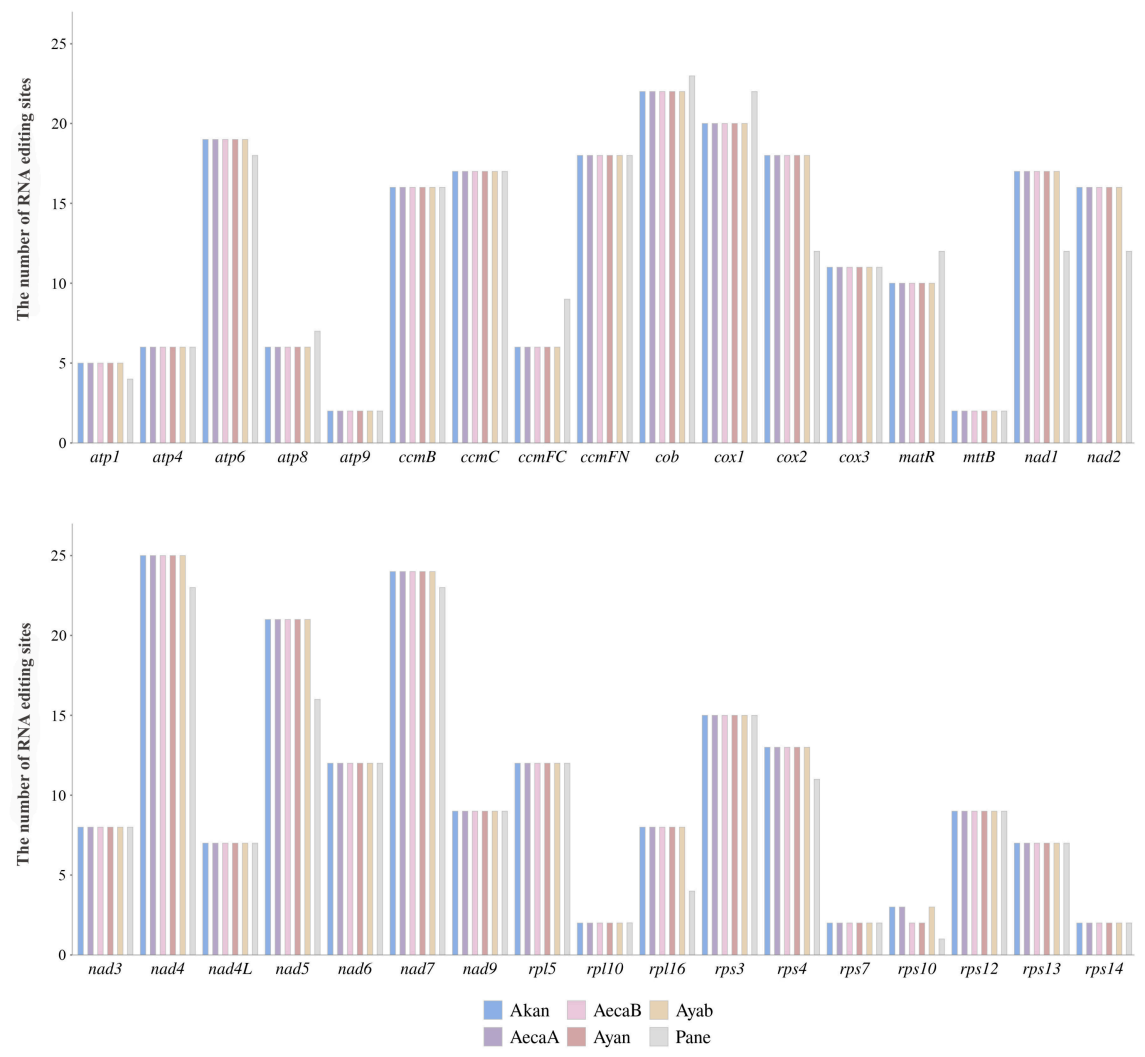
RNA editing numbers of PCGs in the mitochondrial genomes of *A. kansuensis* (Akan), *A. ecalcarata* clade A (AecaA), *A. ecalcarata* clade B (AecaB), *A. yangii* (Ayan), *A. yabeana* (Ayab), and *P. anemonoides* (Pane).

RNA editing sites were predicted for all PCGs across the five mitogenomes and a related species, *P. anemonoides*. The total numbers of predicted editing sites per genome were 393 in *A. kansuensis*, *A. ecalcarata* clade A, and *A. yabeana*; 392 in *A. ecalcarata* clade B and *A. yangii*; and 366 in *P. anemonoides* (Figure 8, Table S10). Most PCGs exhibited identical numbers of predicted editing sites across the five *Aquilegia* mitogenomes. The sole exception was *rps10*, which contained three predicted editing sites in *A. kansuensis*, *A. ecalcarata* clade A and *A. yabeana*, but only two in *A. ecalcarata* clade B and *A. yangii*. The number of predicted editing sites varied substantially among genes, ranging from only two in *atp9*, *rpl10*, *rps14*, and *rps7* to 25 in *nad4* (Figure 8, Table S10). The predicted editing profiles of the *Aquilegia* taxa were generally consistent with that of *P. anemonoides* (Table S10). However, the *Aquilegia* genomes contained 43 lineage-specific predicted RNA editing sites, whereas *P. anemonoides* contains 17 such specific sites (Table S11), with the majority located in *nad* and *rps* genes (Table S11). All predicted editing events occurred at the first or second positions of the codon, with the frequency at the second position being approximately twice that at the first (first position: 127 across all five *Auilegia* taxa; second position: 266 or 265; Table S12). The predicted amino acid changes resulting from predicted RNA editing were grouped into five major categories, among which the conversion from hydrophilic to hydrophobic residues was the most frequent (Table S13). Notably, all RNA editing sites reported in this study were computationally predicted using the PREPACT software, which relies on sequence homology and predefined mitochondrial editing criteria. Although the prediction parameters are well established for plant mitogenomes, experimental validation via RNA-seq or cDNA sequencing is necessary to confirm the actual occurrence and precise efficiency of these editing events.

### 2.6. Phylogenetic Analysis

**Figure 9:**
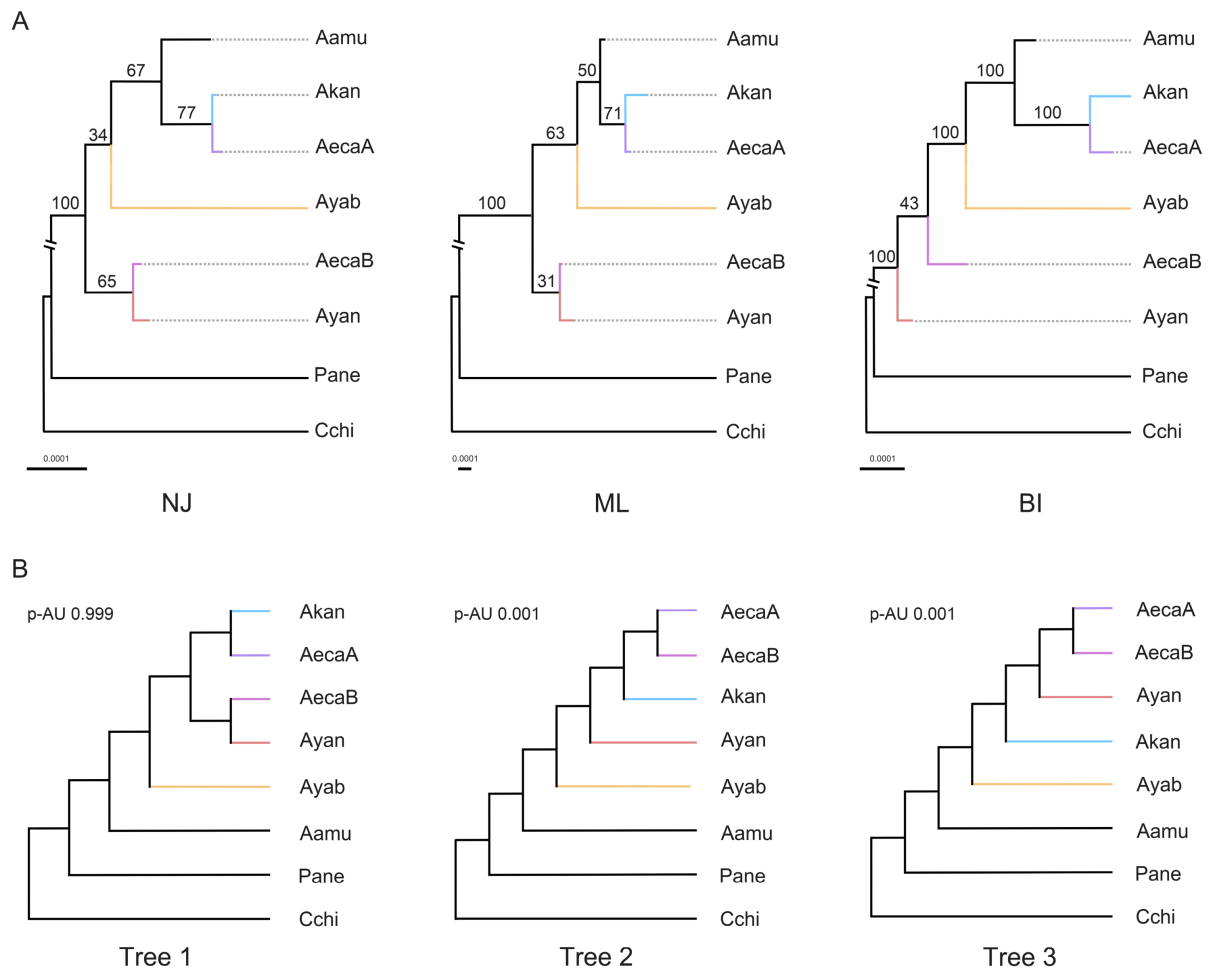
Phylogenetic relationships and topology tests among eight mitochondrial genomes. (**A**) Phylogenetic trees constructed from concatenated sequences of 24 shared PCGs, shown from left to right as neighbor-joining (NJ), maximum likelihood (ML), and Bayesian inference (BI) trees. *C. chinensis* (Cchi) and *P. anemonoides* (Pane) were used as outgroups. Bootstrap values (1000 replicates) are indicated above the branches. (**B**) Results of the AU test comparing the unconstrained topology (Tree 1) with two alternative constrained topologies that force *A. ecalcarata* clade A and clade B to form a monophyletic group (Trees 2 and 3). AU *p*-values (*p*-AU) are shown for each topology.

*A. ecalcarata* clade A and *A. ecalcarata* clade B are both spurless taxa. Although phylogenetic analyses based on nuclear and chloroplast genome data have supported the multiple-origins hypothesis for spurless lineages within the *A. ecalcarata* complex, mitochondrial genome-based evidence has remained lacking. To evaluate this hypothesis from a mitochondrial perspective, we conducted phylogenetic analyses using 24 shared mitochondrial PCGs extracted from five *Aquilegia* taxa, *A. amurensis* (the only published mitochondrial genome within the genus), *P. anemonoides* (outgroup), and *C. chinensis* (outgroup). Following amino acid-guided alignment and trimming, the concatenated nucleotide matrix comprised 16,923 bp, with a high level of completeness (only 0.23%, missing data). However, the matrix was largely invariant: 15,947 (94.23%) sites were constant, whereas only 107 sites (0.63%) were parsimony-informative, indicating limited phylogenetic signal within the dataset. Phylogenetic reconstructions were performed using neighbor-joining (NJ), maximum likelihood (ML), and Bayesian inference (BI). The ML tree was constructed under a gene-partitioned model, with each gene treated as a separate partition. All three methods yielded largely congruent topologies (Figure 9). Within *Aquilegia*, the major relationships involving the spurless lineages were consistently recovered across all analyses. Notably, none of the analyses supported a sister relationship between the two spurless taxa, *A. ecalcarata* clade A and *A. ecalcarata* clade B. Instead, *A. ecalcarata* clade A was consistently grouped with the spurred species *A. kansuensis*, whereas *A. ecalcarata* clade B clustered with another spurred species, *A. yangii*, in both NJ and ML trees. These topologies support the hypothesis that the two spurless lineages are embedded within different evolutionary backgrounds. To statistically test whether *A. ecalcarata* clade A and *A. ecalcarata* clade B share a common spurless ancestor, approximately unbiased (AU) tests were performed by comparing the unconstrained topology with alternative constrained topologies that forced the two clades to form a monophyletic group (Figure S9). The unconstrained reference topology, in which *A. ecalcarata* clade A clustered with *A. kansuensis* and *A. ecalcarata* clade B with *A. yangii*, received strong statistical support (*p*-AU = 0.999). In contrast, both constrained topologies enforcing the monophyly of the two spurless clades were significantly rejected (Figure S9, Tree 2: *p*-AU = 0.001; Tree 3: *p*-AU = 0.001).

### 2.7. Collinearity Analysis Across Five Mitochondrial Genomes

**Figure 10:**
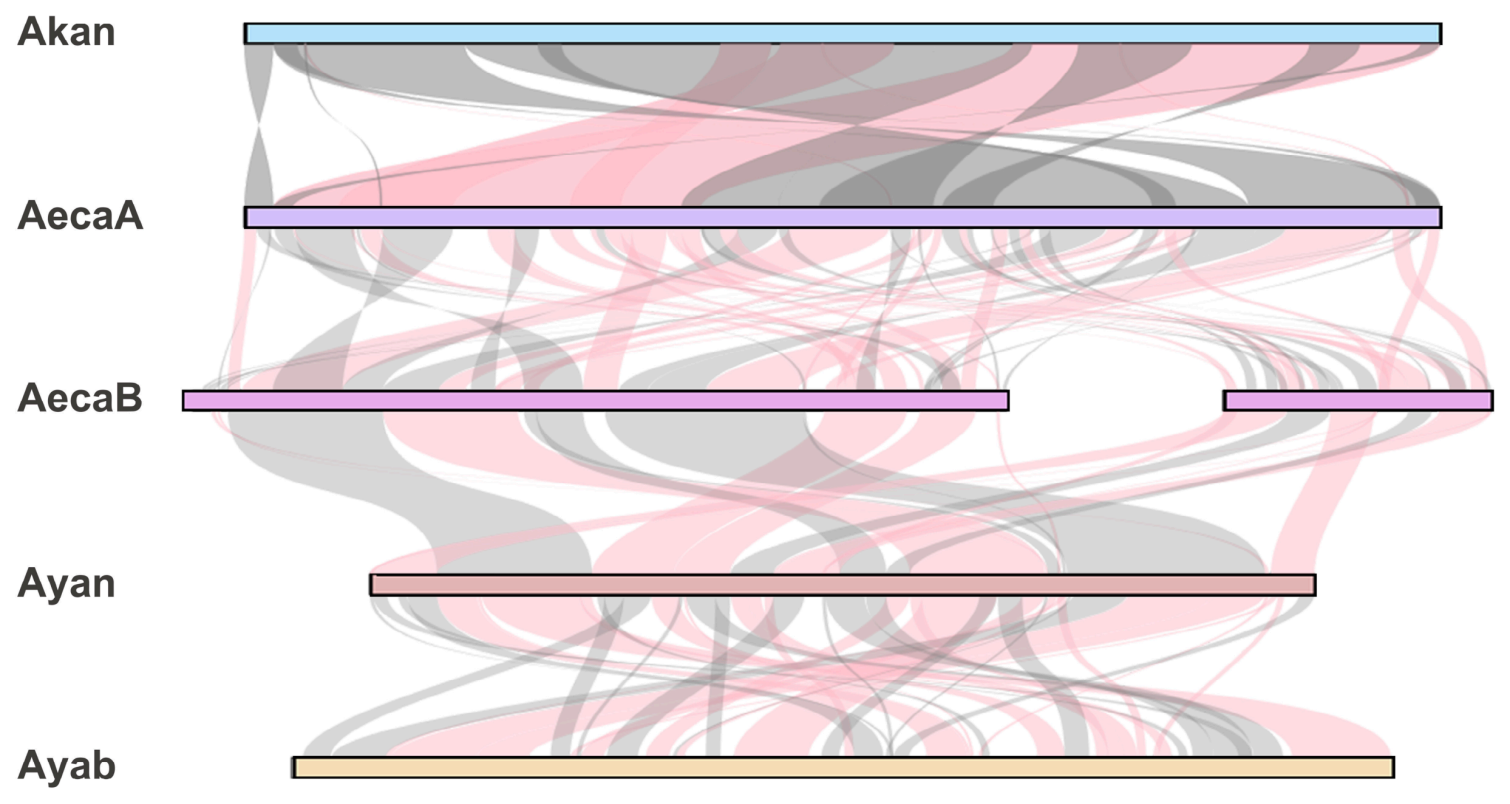
Collinearity analysis of the mitochondrial genomes of *A. kansuensis* (Akan), *A. ecalcarata* clade A (AecaA), *A. ecalcarata* clade B (AecaB), *A. yangii* (Ayan), and *A. yabeana* (Ayab).

Pairwise whole-genome alignment revealed extensive collinear regions among the five mitogenomes, with the degree of conservation clearly reflecting phylogenetic affinity (Figure 10). Strikingly, *A. kansuensis* and *A. ecalcarata* clade A exhibited near-perfect collinearity: only 16 collinear blocks were detected, yet 12 of them exceeded 10 kb and together covered 100% of both genomes (Table S14). This indicates that the mitogenome structures of these two taxa are virtually identical. In contrast, comparisons involving *A. ecalcarata* clade B, *A. yangii*, and *A. yabeana* revealed more fragmented collinearity. For instance, *A. ecalcarata* clade A and *A. ecalcarata* clade B shared 65 collinear blocks, of which 14 were larger than 10 kb, covering 91.02% and 84.46% of the two genomes, respectively (Table S14). Similarly, *A. ecalcarata* clade B and *A. yangii* shared 21 blocks (11 > 10 kb, covering 87.52% and 98.74%), and *A. yangii* and *A. yabeana* shared 41 blocks (17 > 10 kb, covering 95.94% and 82.47%) (Table S14). The higher number of collinear blocks and incomplete genome coverage in these comparisons suggest that the mitogenomes of *A. ecalcarata* clade B, *A. yangii*, and *A. yabeana* experienced more extensive structural rearrangements (Table S14).

## 3. Discussion

### 3.1. The Structures of Five Mitochondrial Genomes

Our third-generation sequencing-based assemblies uncovered complex structural configurations in the mitochondrial genomes of the *A*. *ecalcarata* complex. Based on contig connectivity and average sequencing depth, the genomes of *A. kansuensis*, *A. ecalcarata* clade A, and *A. yangii* were resolved as a single circular molecule, whereas *A. ecalcarata* clade B yielded a dual circular structure. For *A. yabeana*, the assembly graph revealed a complex topology that could not be resolved into a simple circular form; however, to facilitate clear presentation and subsequent analyses, we have represented it as a linear molecule. Early studies proposed that plant mitochondrial DNA could be represented as a single circular master chromosome [9,32], a view that dominated the field for decades. However, this paradigm has been revised by accumulating evidence for diverse mitogenome architectures [33,34,35,36,37]. For instance, the mitochondrial genome of *Silene noctiflora* comprises more than 50 circular chromosomes [38], and *Lactuca sativa* mitochondria simultaneously display linear, circular, and branched conformations [39]. It is now widely recognized that plant mitogenomes exist as dynamic, multi-structural populations, including circular, linear, branched, multimeric circular, and branched circular molecules. Critically, the assemblies present here were constructed based on contig connectivity and average sequencing depth; although third-generation sequencing provides richer structural information than short-read approaches, these assemblies are computational representations and do not directly reveal the in vivo physical organization.

Long repetitive sequences are a common feature of plant mitochondrial genomes and are well known to mediate structural variation via homologous recombination [5]. In this study, long repetitive sequences were detected in all five taxa. In the assembly graphs, these repeats often manifested as duplicate contig pairs, further implying the potential coexistence of multiple structural isoforms. Specifically, palindromic repeats can mediate inversions of the intervening sequence, whereas direct repeats mediate the transition from a master circular structure to two subgenomic circular molecules (Supplementary File S3). Sequencing reads provide support for alternative genomic structures mediated by long repetitive sequences across all five taxa (Supplementary File S3). Future investigations employing complementary approaches such as fluorescence microscopy, transmission electron microscopy, and gel electrophoresis will be required to elucidate the native configurations of these mitogenomes.

### 3.2. tRNA Sets in Five Mitochondrial Genomes

Eukaryotic mitochondria originated from an α-proteobacterial endosymbiont and retain a compact genome and translation machinery [40,41]. For more than 2 billion years, mitochondrial genes have co-evolved with the nuclear genome, during which most ancestral mitochondrial genes have been lost or transferred to the nucleus, leaving a reduced set of genes encoding oxidative phosphorylation subunits, rRNA, and a variable complement of tRNAs [42,43,44]. Among these, tRNA gene content exhibits the greatest variation: the *Chlamydomonas reinhardtii* mitogenome encodes only three tRNA genes [45], whereas that of *Amborella trichopoda* harbors over 150 [46]. The five taxa of the *A. ecalcarata* complex retain 20–23 unique tRNA genes, comparable to the 22 unique tRNA genes reported in *A. amurensis* [47], suggesting conserved tRNA gene content within the genus. In addition to ancestral gene loss, plant mitogenomes frequently acquire sequences from plastids, the nucleus, and even exogenous sources [48], thereby diversifying the mitochondrial tRNA pool. Our data provide clear evidence that chloroplast-derived fragments have contributed to the mitochondrial tRNA sets in all five taxa (Table S7, Figure S8). The tRNA genes *trnH-GUG*, *trnN-GUU*, *trnM-CAU*, *trnD-GUC, trnP-UGG*, and *trnW-CCA* were identified in chloroplast–mitochondrial collinear blocks in every taxon, indicating that the transfer of all six tRNA genes from the chloroplast to the mitochondrion represents an ancestral event predating the divergence of these lineages. The tRNA genes *trnA-UGG*, *trnI-GAU*, and *trnV-GAC* are present only in MTPTs shared between *A. ecalcarata* clade B and *A. yangii* (Table S7, Figure S8). The tRNA gene *trnL-CAA* was identified on the MTPTs shared between *A. ecalcarata* clade A and *A. kansuensis* (Table S7, Figure S8). Collectively, these findings indicate that the chloroplast-to-mitochondrial transfer of *trnH-GUG*, *trnN-GUU*, *trnM-CAU*, *trnD-GUC*, *trnP-UGG*, and *trnW-CCA* represents a shared ancestral event across the *A*. *ecalcarata* complex. An additional gain of *trnA-UGG*, *trnI-GAU*, and *trnV-GAC* is retained exclusively in *A. ecalcarata* clade B and *A. yangii*, whereas *trnL-CAA* represents a separate transfer event shared by *A. ecalcarata* clade A and *A. kansuensis* (Table S7, Figure S8).

### 3.3. Discordance Between Codon Usage Bias and the Mitochondrial tRNA Set

During translation, tRNAs serve as essential adaptors that deliver specific amino acids in response to mRNA codons. The composition and abundance of the tRNA pool are therefore expected to co-evolve with codon usage bias [49,50]. Intriguingly, our analysis revealed a marked discordance between the codon preferences of mitochondrial PCGs and the encoded tRNA set. For example, *A. ecalcarata* clade B and *A. yangii* have the single-copy tRNA (*trnA-UGC*) to transfer alanine with a moderate RSCU of 1.00 or 0.99 (Table S8), whereas the *trnA* gene is absent from the mitogenomes of the other three taxa (Table S3). Similarly, all five mitogenomes encode *trnY-GUA,* yet its cognate codon UAC has an RSCU of only 0.51, while the preferred tyrosine codon UAU (RSCU = 1.49) lacks a corresponding mitochondrial tRNA (Tables S3 and S8). At least two mechanisms can account for this apparent inconsistency. First, the import of nuclear-encoded tRNA is a well-documented compensatory strategy in plant mitochondria [51,52]. Although *A. ecalcarata* clade A, *A. kansuensis*, and *A. yabeana* lack *trnA*, the mitochondrial translation machinery still requires alanine; nuclear-encoded tRNA^Ala^ imported from the cytoplasm likely compensates for this absence. Second, wobble and super-wobble pairing [53,54,55,56] allows a single tRNA species to decode multiple synonymous codons through non-canonical base pairing between the anticodon and the codon. For example, a single plastid-encoded tRNA^Gly(UCC)^ is sufficient to decode all four glycine codons (GGNs) in tobacco chloroplasts [55]. The observed codon preferences of mitochondrial PCGs therefore reflect the integrated effects of the mitochondrially encoded tRNA set, cytosolic tRNA import, and the decoding capacity enabled by wobble pairing, rather than the mitochondrial tRNA repertoire alone.

### 3.4. Twice-Independent Origins of Spurless A. ecalcarata

The *A*. *ecalcarata* complex is an exceptional model for studying the parallel evolution of phenotypic traits [17,18]. Multiple molecular phylogenetic studies have consistently recovered the morphologically defined *A. ecalcarata* as a non-monophyletic species, providing strong evidence that the spurless phenotype originated on at least two independent occasions [26,27,28,29,30,31]. A recent phylogenetic study has revealed introgression and incomplete lineage sorting in the evolutionary history of the *A. ecalcarata* complex, leading to widespread phylogenetic conflicts. To address this, Liu et al. [31] applied strict thresholds to exclude data from hybrid individuals and constructed a coalescent phylogenetic tree using resequencing data of nuclear genomes from 35 pure-line individuals. This coalescent tree supports the hypothesis that *A. ecalcarata* originated twice independently. However, all published phylogenetic studies were based on the nuclear or chloroplast data. Our study provides the first mitochondrial genome-based perspective on relationships within this complex.

In our mitochondrial phylogeny, several internal nodes showed weak support and unstable relationships (Figure 9 and Figure S9). The limited resolution is likely associated with the restricted phylogenetic information available in mitochondrial sequences. In our dataset, 94.23% of nucleotide positions were constant, while only 107 sites were parsimony informative. Moreover, phylogenetic analyses partitioned by codon positions demonstrated heterogeneous phylogenetic signals (Figure S9). While the overall number of parsimony-informative sites is low, they nonetheless provide sufficient signal for resolving the deep, major lineage divergences within the complex, though power is limited for more recent diversification events. The placement of *A. amurensis* within the complex may also reflect the limited number of available reference genomes and the absence of closely related intermediate lineages. In addition, mitochondrial relationships may not always fully reflect species-level evolutionary histories, because mitochondrial genomes primarily represent maternal evolutionary histories. Therefore, the precise placement of *A. amurensis* requires further investigation using broader sampling and nuclear genomic data.

Despite the uncertainty in some internal relationships, all phylogenetic analyses (ML, NJ, and BI) consistently recovered *A. ecalcarata* clade A and *A. ecalcarata* clade B as non-sister lineages (Figure 9A). Furthermore, AU tests significantly rejected alternative topologies in which *A. ecalcarata* clade A and *A. ecalcarata* clade B were constrained as sister taxa (Figure 9B). Therefore, the uncertainty in the deeper relationships within the *Aquilegia* complex does not affect the conclusion that the two spurless lineages did not originate from a common ancestor. This conclusion is further supported by multiple independent mitogenomic features, including the near-perfect synteny between the *A. kansuensis* and *A. ecalcarata* clade A genomes, the shared possession of *trnA-UGG*, *trnI-GCC*, and *trnV-GAC* in *A. ecalcarata* clade B and *A. yangii* (Table S1). Collectively, these mitochondrial genomic signatures provide independent evidence that the two spurless lineages are separated by spurred taxa and represent independent origins of the spurless morphotype, consistent with previous nuclear genome-based conclusions [31]. However, as our analysis was based on only five individuals, further population-level analyses are necessary to determine whether this evolutionary pattern is fixed across the entire complex from a mitochondrial perspective.

## 4. Materials and Methods

### 4.1. Plant Material Sampling and Sequencing

Sampling was guided by the phylogenetic framework established by Liu et al. [31], which recovered three major clades within the *A. ecalcarata* complex: two internal clades (clade A and clade B) and *A. yabeana* as their sister lineage. In that study, clade A comprises *A. kansuensis* and a subset of *A. ecalcarata* individuals, while clade B contains *A. yangii*, *A. rockii*, and the remaining *A. ecalcarata* individuals, along with additional *A. kansuensis* accessions. In this study, one individual of *A. kansuensis* in clade A was collected to represent the spurred lineage that is sister to *A. ecalcarata* clade A in our mitochondrial dataset. Two *A. ecalcarata* individuals, one from each clade, were included to capture both independent origins of the spurless morphotype. *A. yangii* and *A. yabeana* were sampled as additional representatives of the complex. Fresh leaves of *A. ecalcarata* clade A (Taibai, Shaanxi, China), *A. ecalcarata* clade B (Jiulong, Sichuan, China), *A. kansuensis* (Wuxi, Chongqing, China), and *A. yabeana* (Baihuashan, Beijing, China) were collected. Total genomic DNA was extracted using the cetyltrimethylammonium bromide (CTAB) method. The extracted DNA samples were processed following the standard PacBio SMRTbell adapter protocol (BioMarker, Beijing, China), including DNA assessment, DNA fragmentation, end repair, SMRTbell adapter ligation, exonuclease treatment, size selection and library quality assessment. Qualified libraries were sequenced on the PacBio Sequel II platform (BioMarker, Beijing, China).

### 4.2. Mitochondrial Genome Assembly

For *A. kansuensis*, *A. ecalcarata* clade A, *A. ecalcarata* clade B, and *A. yabeana*, assemblies were generated from unpublished PacBio HiFi data. For *A. yangii*, we used published PacBio long-read data (NCBI BioProject: PRJNA636213), originally deposited under the name *Aquilegia oxysepala* var. *kansuensis* [57]. All five mitochondrial genomes were initially assembled using PMAT2 [58]. To validate assembly accuracy, raw reads were mapped back to the assemblies using Minimap2 [59]. In two species, *A. yangii* and *A. yabeana*, some contig junctions identified in the initial assemblies were not supported by long spanning reads. These two genomes were therefore reassembled using alternative assemblers (Oatk and HiMT) [60,61], and the assembly with the strongest read-mapping support was retained for downstream analyses. The final assemblies were generated as follows: *A. kansuensis*, *A. ecalcarata* clade A, and *A. ecalcarata* clade B were assembled using PMAT (v2.0) [58] in “autoMito” mode with parameters “-g 300m -t hifi”; *A. yangii* was assembled using HiMT (v 1.1.1) [61] with parameters “-d CLR”; and *A.yabeana* was assembled using Oatk (v1.0) [60] with parameters “-k 1001 -c 250.” Assembly graphs were visualized using Bandage (v0.8.1) [62], and structural optimization was performed based on the average depth and connectivity of contigs.

### 4.3. Gene Annotation

Annotation of protein-coding genes (PCGs) was performed using two methods: the online software GeSeq (https://chlorobox.mpimp-golm.mpg.de/geseq.html, accessed on 24 March 2026) and PMGA (http://www.1kmpg.cn/pmga/, accessed on 25 March 2026). For GeSeq annotation [63], the mitochondrial genome of *A. amurensis* was used as the reference. For PMGA annotation [64], prediction used the plant mitogenome database “29 mitogenomes” as a reference. The annotation results from both software tools were integrated, and final annotations were obtained through manual curation with emphasis on the verification of start and stop codons. Putative pseudogenes were identified based on the position and type of premature termination codons (PTCs) in predicted genes, and further validated by comparison with functional homologs in mitogenomes of closely related species. Annotation of ribosomal RNA (rRNA) and transfer RNA (tRNA) genes was performed using PMGA. Mitochondrial genome maps were visualized using OGDRAW (https://chlorobox.mpimp-golm.mpg.de/OGDraw.html, accessed on 10 April 2026) [65]. Assembled and annotated sequences have been deposited in the GenBank database.

### 4.4. Chloroplast-Derived Fragment Analysis

Chloroplast genomes of five *Aquilegia* were assembled using PMAT (v2.0) [58] with the parameter “-g 300m -t hifi -G pt” for *A. kansuensis*, *A. ecalcarata* clade A, *A. ecalcarata* clade B, and *A. yabeana* and the parameter “-g 300m -t clr -S canu -C canu -G pt” for *A. yangii.* All chloroplast genomes were annotated using GeSeq [63] using “MPI-MP chloroplast reference set” as a reference. Homologous fragments between chloroplast and mitochondrial genomes were identified using BLASTN, with a percentage identity parameter of 70% and an E-value parameter of 1 × 10^−5^. The MTPTs were identified to be either shared or lineage-specific using BLASTN, with a percentage identity parameter of 80%, a length limit of 30, and an E-value parameter of 1 × 10^−5^. The resulting homology relationships were visualized using Circos (v0.69) [66].

### 4.5. Codon Usage Bias Analysis

Codon usage bias was analyzed using CodonW (v1.4.4). A custom script was used to summarize the relative synonymous codon usage (RSCU) for each protein-coding gene. The theoretical effective number of codons (ENC) value was calculated according to the formula ENC_exp_ = 2 + GC3 + 29/[GC3^2^ + (1 − GC3)^2^]. The ENC ratio was calculated according to the formula ENC ratio = (ENC_exp_ − ENC_act_)/ENC_exp_. The RSCU distribution and the ENC plots were visualized using custom R scripts.

### 4.6. Repetitive Sequence Analysis

Simple sequence repeats (SSRs) in the mitochondrial genomes of five taxa were identified using the MISA online tool (http://misaweb.ipk-gatersleben.de/, accessed on 8 May 2026) [67]. Parameters were set as follows: Minimum repeat counts for mono-nucleotide, dinucleotide, trinucleotide, tetranucleotide, pentanucleotide, and hexanucleotide repeats were 10, 5, 4, 3, 3, and 3, respectively. The maximum interval length between adjacent SSRs was set to 0 bp. Dispersed repeat sequences were detected using Vmatch (v2.3.1) (https://github.com/genometools/vstree/, accessed on 9 May 2026, written by Stefan Kurtz), with a Hamming distance of 3 and a minimum repeat length of 30 bp.

### 4.7. RNA Editing Site Analysis

RNA editing sites were predicted using the online tool PREPACT (v3.0) [68]. C-to-U editing events were identified via the BLASTX module (v2.5.0), with Vitis vinifera as the reference and an E-value threshold of 0.001. Prediction results were visualized using a custom R script.

### 4.8. Phylogenetic Analysis

A concatenated supermatrix of 16,923 bp was assembled from 24 PCGs shared by the five newly assembled mitochondrial genomes and *Aquilegia amurensis*, the only previously published mitochondrial genome in the genus *Aquilegia*, with *C. chinensis* and *P. anemonoides* designated as outgroups. Amino acid sequences of the 24 PCGs were aligned using MAFFT v7.525 [69] and trimmed with trimAl v1.5 [70]; the trimmed alignments were then used to guide codon-based nucleotide sequence alignments, which were concatenated into a single supermatrix.

Phylogenetic trees were reconstructed using NJ, ML, and BI methods. The NJ tree was constructed in MEGA12 [71] using the *p*-distance model, incorporating both transitions and transversions, with branch support assessed via 1000 bootstrap replicates. ML analyses were performed with IQ TREE v3.0.1 [72] under the codon substitution model (-st CODON). The optimal partitioning scheme and substitution models were selected by ModelFinder Plus based on the Bayesian Information Criterion (BIC). Six partitions were defined, with the following best-fit models: 

Partition 1 (*atp1* + *mttB* + *nad6* + *rpl15* + *rps10* + *rps13* + *rps14*, 1463 bp, MG + F1X4 + R3 model); Partition 2 (*atp4* + *atp6* + *atp8* + *ccmB* + *cob* + *cox1* + *cox3* + *nad2* + *nad3* + *nad4* + *nad9* + *rpl16* + *rps12*, 3268 bp, MG + F3X4 + I model); Partition 3 (*atp9*, 72 bp, KOSI07 + F1X4 + R2 model); Partition 4 (*ccmC*, 315 bp, KOSI07 + F1X4 + I + G4); Partition 5 (*nad7*, 384 bp, SCHN05 + F3X4 + I + G4 model); and Partition 6 (*rps7*, 139 bp, KOSI07 + F1X4 model). 

All branch supports in the ML tree were evaluated with 1000 ultrafast bootstrap replicates. Additionally, separate ML analyses were conducted for each of the three codon positions independently using the same partitioning strategy. BI analysis was carried out in MrBayes v3.2.7 [73] under the HKY + F + I model (lset nst = 2 rates = propinv). Two independent MCMC runs were performed, each with four chains for 2,000,000 generations, sampling every 1000 generations, and the first 25% of samples were discarded as burn-in before constructing the consensus tree.

To evaluate whether the two spurless lineages formed a monophyletic group, an AU test was conducted in IQ-TREE with 10,000 bootstrap replicates. One unconstrained reference topology and two constrained topologies enforcing *A. ecalcarata* clade A and *A. ecalcarata* clade B as a monophyletic group were compared to assess the statistical significance of this branch hypothesis.

### 4.9. Collinearity Analysis Across Five Taxa

The collinearity analysis across the five mitogenomes was performed using BLASTN, with an E-value of 10^−5^ and a minimum sequence identity of 70%. Collinear blocks longer than 1000 bp were selected, and a multiple collinearity map was generated using LINKVIEW2 (v1.0.5) (https://github.com/YangJianshun/LINKVIEW2, accessed on 10 May 2026) to visualize structural conservation and rearrangement patterns among mitochondrial genomes.

## 5. Conclusions

This study presents the first mitochondrial genomes for five taxa within the *A. ecalcarata* complex: *A. kansuensis*, *A. ecalcarata* clade A, *A. ecalcarata* clade B, *A. yangii*, and *A. yabeana*. Phylogenetic analysis based on mitochondrial data consistently placed the two spurless *A. ecalcarata* samples in separate clades. This topology, together with complementary evidence from genome collinearity, tRNA gene content, and MTPT patterns, supports the independent origins of the spurless morphotype and corroborates the non-monophyly of the morphologically defined *A. ecalcarata*. Our results are consistent with the parallel evolution of the spurless morphotype and provide organellar evidence that can inform future taxonomic work on these independently derived lineages. The extensive structural heterogeneity observed among these mitogenomes underscores the need for further methodological development to better resolve and integrate highly rearranged mitochondrial genomes into population-level analyses. This study provides the first mitochondrial genetic resource for future organellar-level phylogenetic and evolutionary investigations of the *A. ecalcarata* complex.

## Figures and Tables

**Table 1:** Summary of assembly statistics.

	*A. kansuensis*	*A. ecalcarata* Clade A	*A. ecalcarata* Clade B	*A. yangii*	*A. yabeana*
Genome length (bp)	618,203	618,154	559,318	488,419	568,238
GC (%)	46.31	46.31	45.98	46.31	45.99
Number of genes	79	79	74	66	66
Number of PCGs	40 (35)	40 (35)	35 (35)	35 (35)	36 (35)
Number of tRNA genes	34 (21)	34 (21)	34 (23)	28 (21)	27 (20)
Number of rRNA genes	5 (3)	5 (3)	5 (3)	3 (3)	3 (3)
Total length of genes	48,107	48,107	39,755	38,779	38,940
Total length of PCGs	35,592	35,592	30,867	30,621	30,840
Total length of tRNA genes	2547	2547	2576	2082	2023
Total length of rRNA genes	9968	9968	6312	6076	6077

Number of PCGs, tRNA genes, and rRNA genes are presented as total number (number of unique genes after removing duplicates).

## Data Availability

DNA sequences of the five mitogenomes and chloroplast genomes of the *A. ecalcarata* complex have been deposited in the GenBank database under the following accession numbers, and are part of BioProject PRJNA1492311: *Aquilegia ecalcarata* clade A mitochondrial genome: PZ692395; *Aquilegia ecalcarata* clade B mitochondrial genome: PZ704477-PZ704478; *Aquilegia kansuensis* mitochondrial genome: PZ692394; *Aquilegia yangii* mitochondrial genome: PZ692397; *Aquilegia yabeana* mitochondrial genome: PZ692396; *Aquilegia ecalcarata* clade A chloroplast genome: PZ706439; *Aquilegia ecalcarata* clade B chloroplast genome: PZ706443; *Aquilegia kansuensis* chloroplast genome: PZ706446; *Aquilegia yangii* chloroplast genome: PZ706445; *Aquilegia yabeana* chloroplast genome: PZ706444. The raw PacBio HiFi sequencing reads used for genome assembly are not publicly available at this time because they are the primary data for an ongoing nuclear genome project (manuscript in preparation). These raw data will be released upon publication of the nuclear genome. Until then, the raw sequencing reads are available from the corresponding author upon reasonable request. The scripts used are available on Github (https://github.com/xiaoyaoximena/Assembly-and-comparative-analysis-of-Aquilegia-ecalcarata-complex, accessed on 18 July 2026).

## Supplementary Materials

The following supporting information can be downloaded at: https://www.mdpi.com/article/10.3390/ijms27156589/s1.

## Abbreviations

The following abbreviations are used in this manuscript:

MTPTs	Mitochondrial–plastid DNAs
RSCU	Relative synonymous codon usage
PCGs	Protein-coding genes
