# Supplementary material for: Assembly and Comparative Analysis of Five Complete Mitochondrial Genomes of the Aquilegia ecalcarata Complex
Source: Int J Mol Sci. 2026 Jul 24;27(15):6589. doi: 10.3390/ijms27156589 (PMC13464993; doi:10.3390/ijms27156589)
Supplement: Supplementary file 1 [file ijms-27-06589-s001.zip › Supplementary file S2_Figures.pdf]

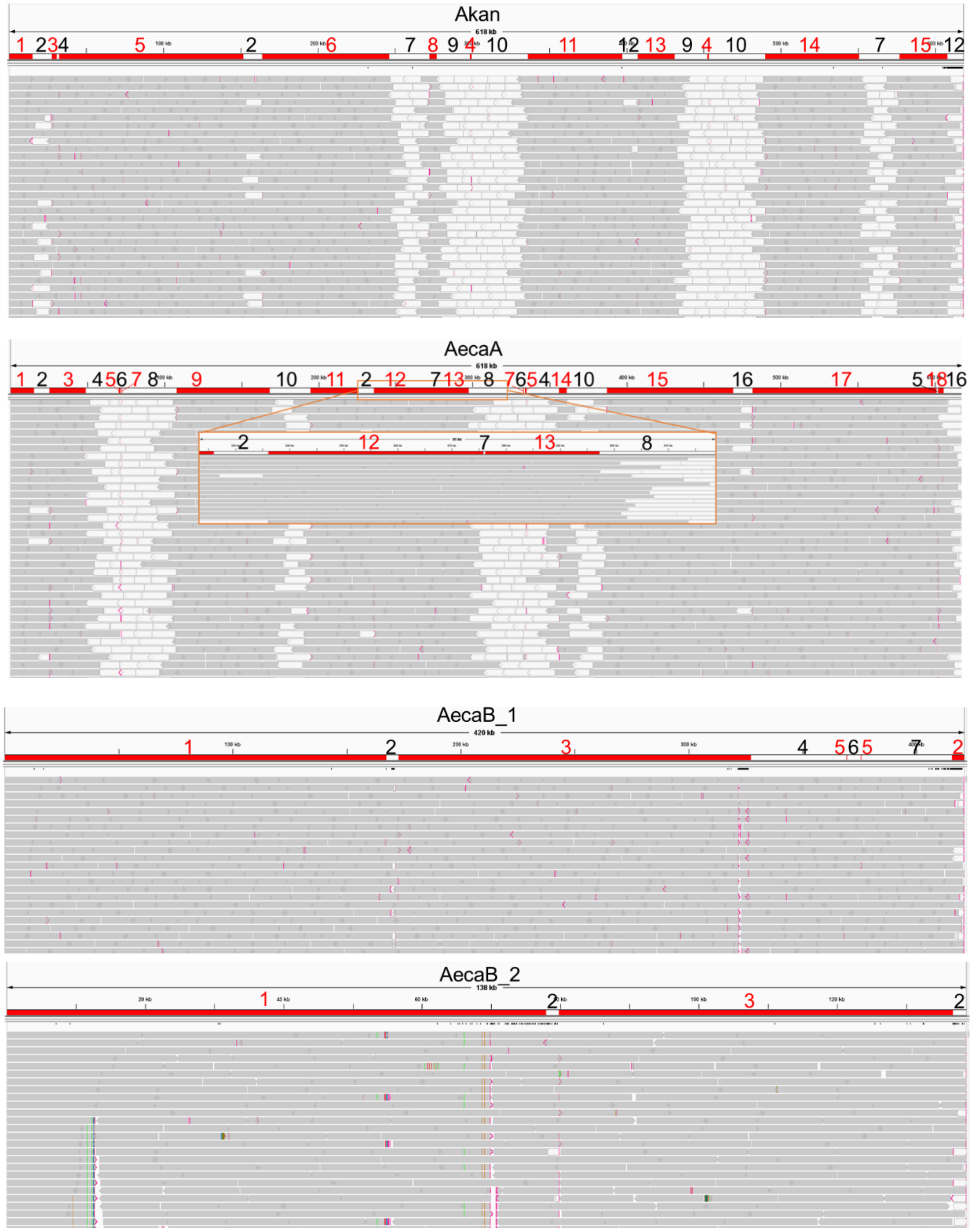
Akan
AecaA
AecaB_1
AecaB_2

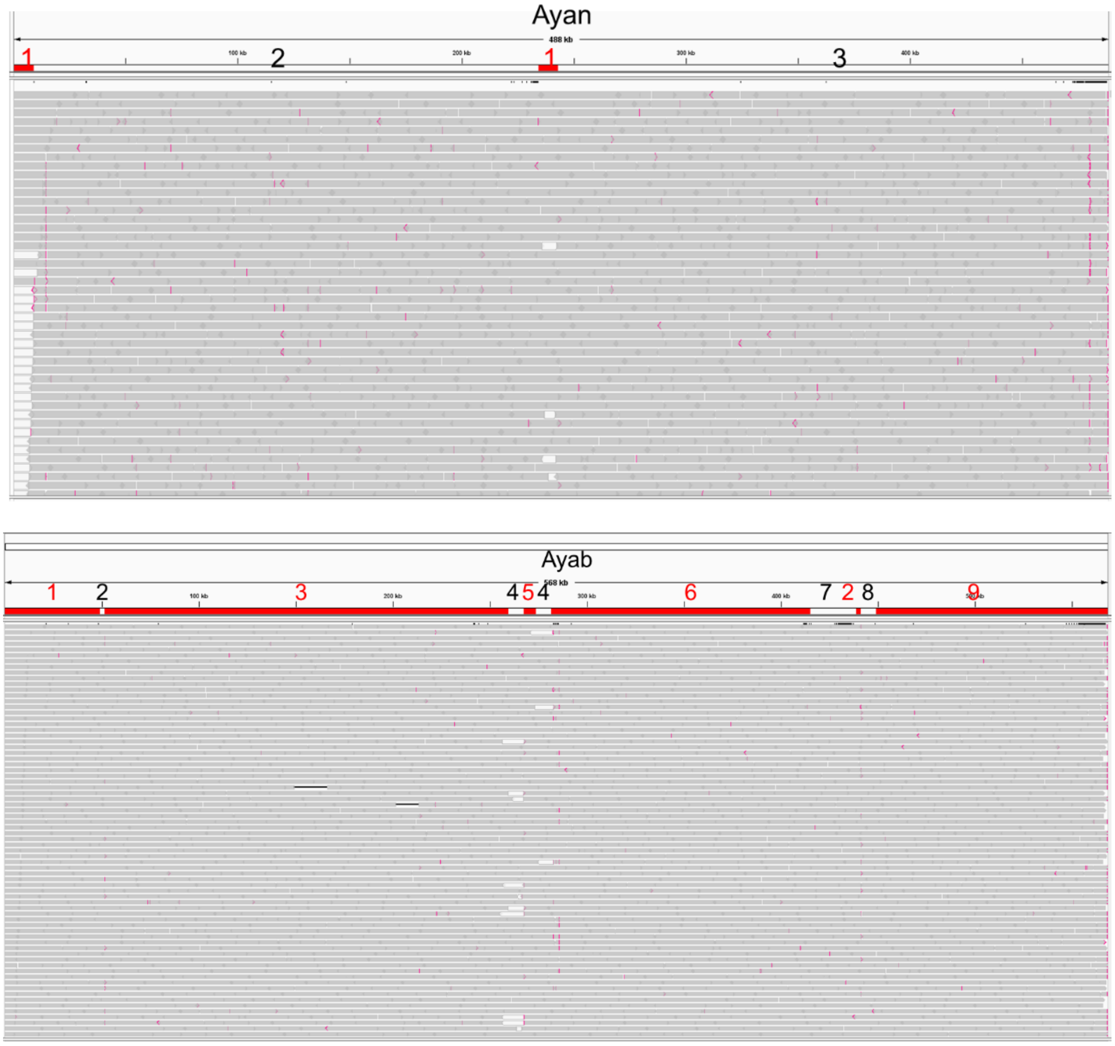

Figure S1 IGV presentation of raw reads mapped on assembled mitogenomes of *A. kansuensis* (Akan), *A. ecalcarata*_clade A (AecaA), *A. ecalcarata*_clade B (AecaB), *A. yanggi* (Ayan), and *A. yabeana* (Ayab). Alternating red and white markers indicate contig positions, and corresponding contig-IDs are annotated above for clear identification. White reads are primarily caused by multiple alignments resulting from long repetitive sequences.

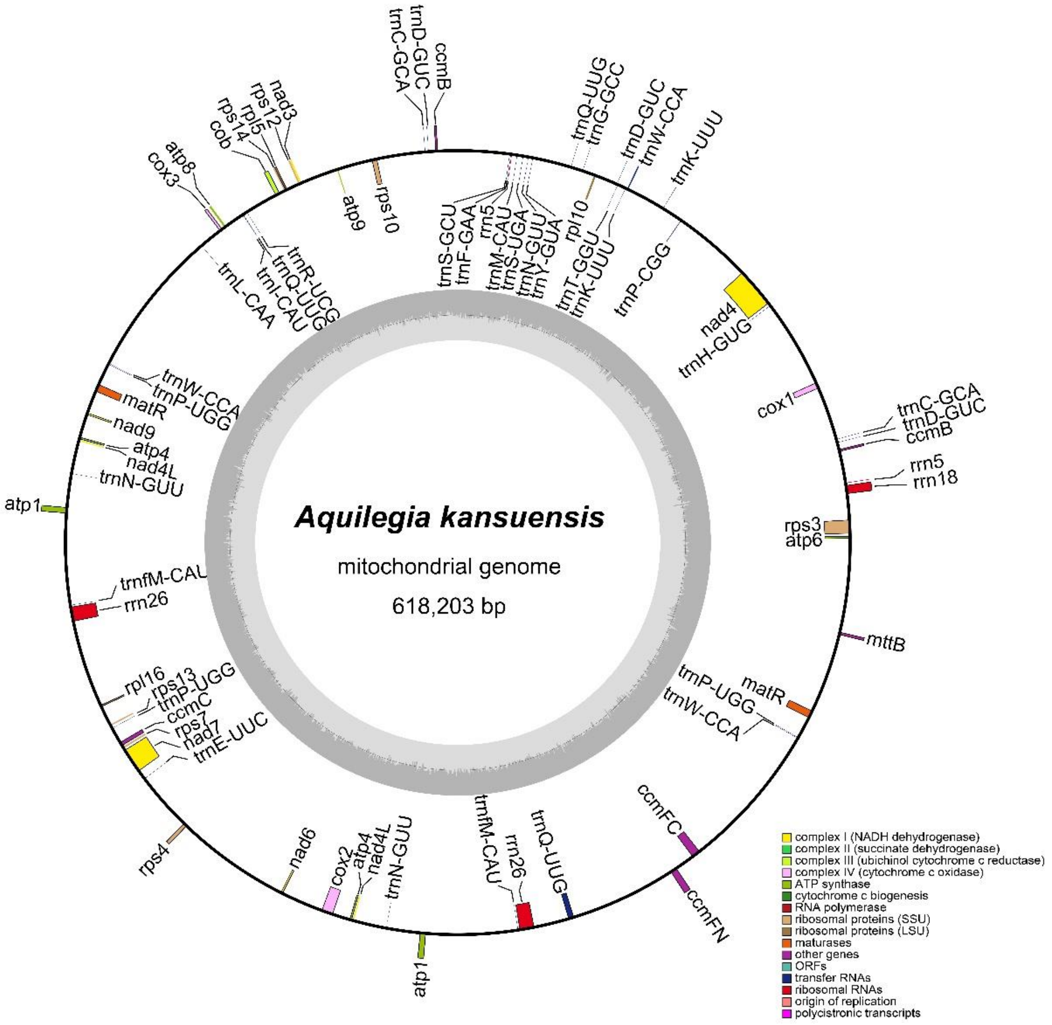

Figure S2 Schematic representation of the mitochondrial genomes of *A. kansuensis*. Genes located on the outside of the circle are on the forward strand, whereas the genes inside circle are on the reverse strand. The genes are color-grouped according to functions.

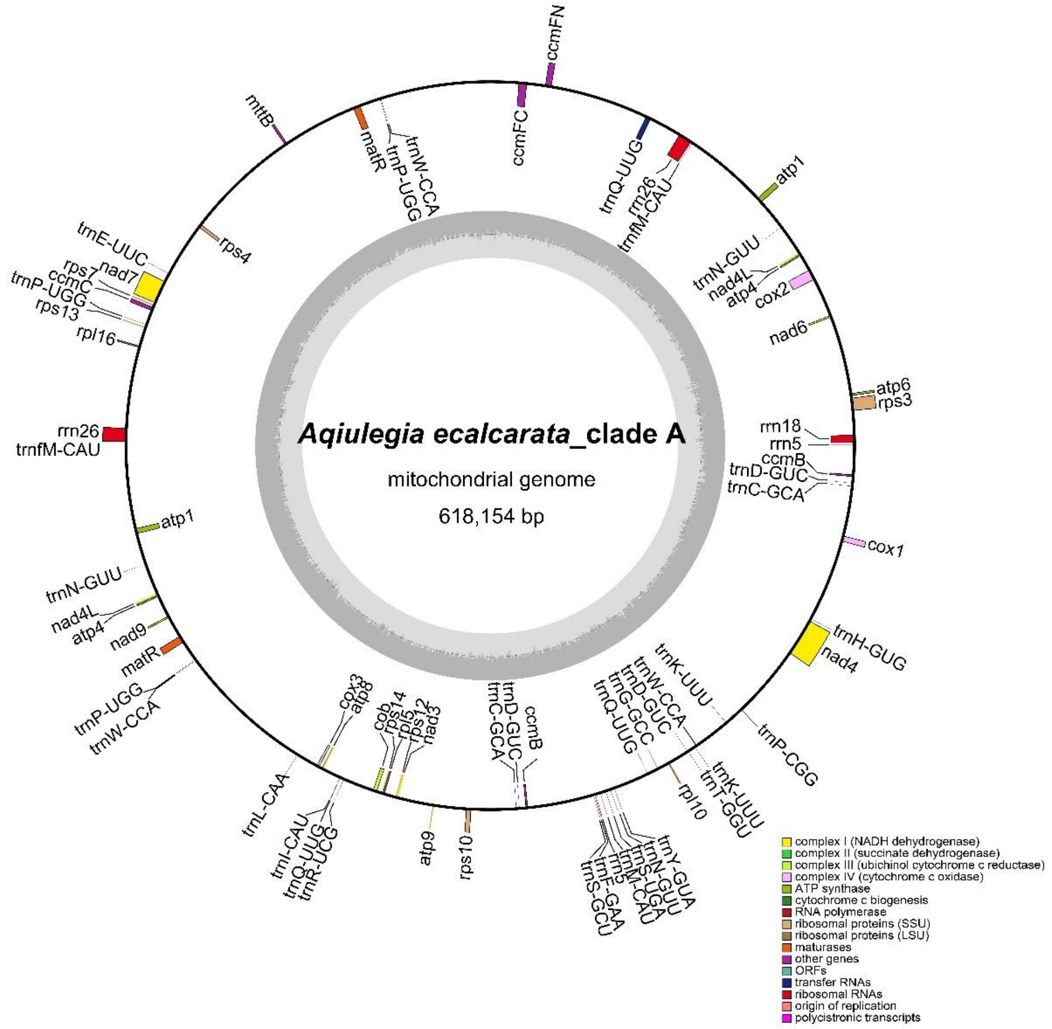

Figure S3. Schematic representation of the mitochondrial genomes of *A. ecalcarata*_clade A. Genes located on the outside of the circle are on the forward strand, whereas the genes inside circle are on the reverse strand. The genes are color-grouped according to functions.

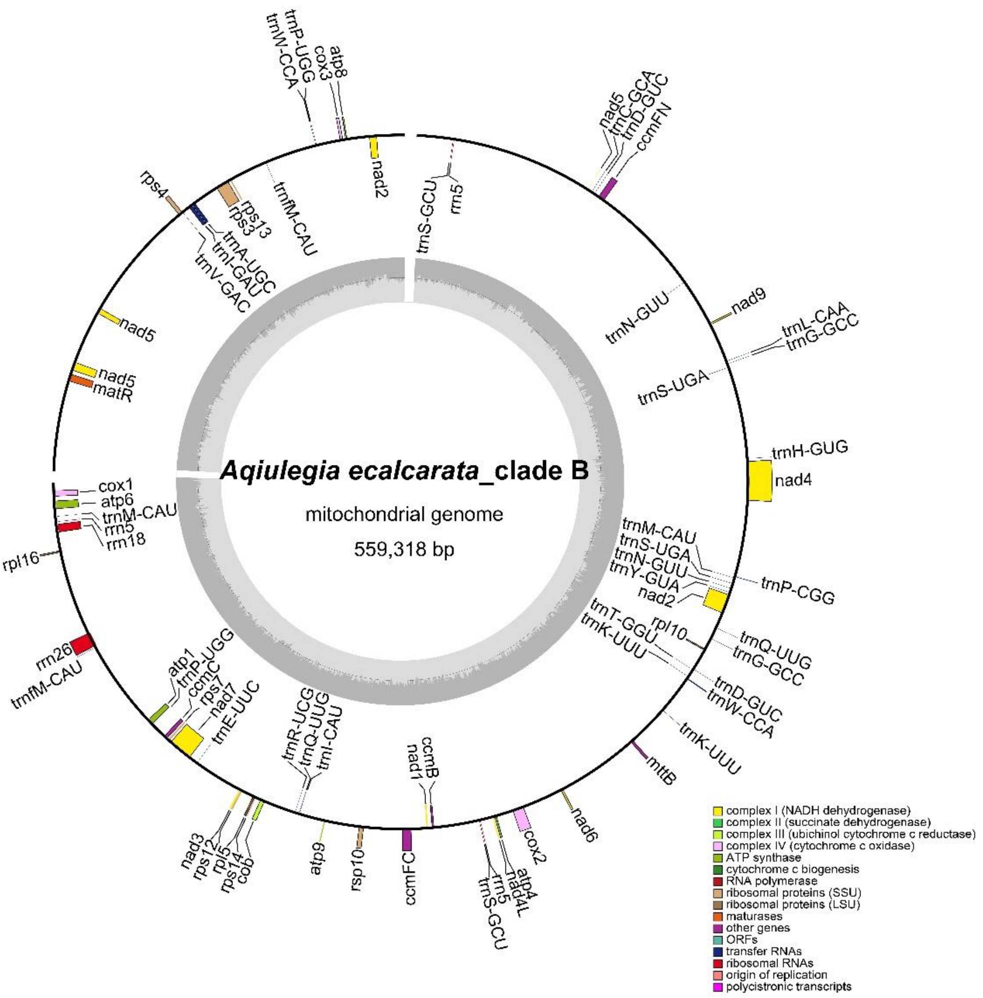

Figure S4. Schematic representation of the mitochondrial genomes of *A. ecalcarata*_clade B. Genes located on the outside of the circle are on the forward strand, whereas the genes inside circle are on the reverse strand. The genes are color-grouped according to functions.

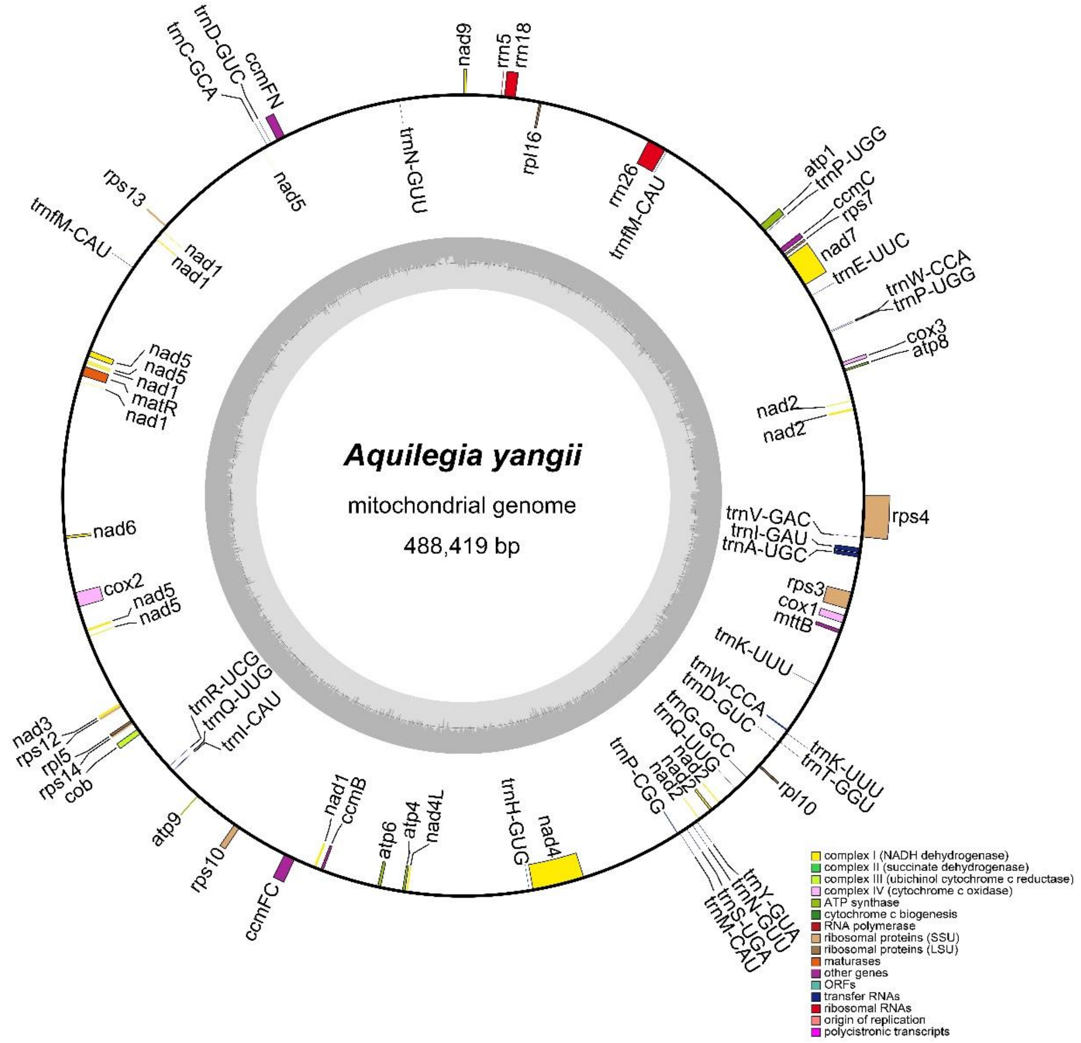

Figure S5. Schematic representation of the mitochondrial genomes of *A. yangii*. Genes located on the outside of the circle are on the forward strand, whereas the genes inside circle are on the reverse strand. The genes are color-grouped according to functions.

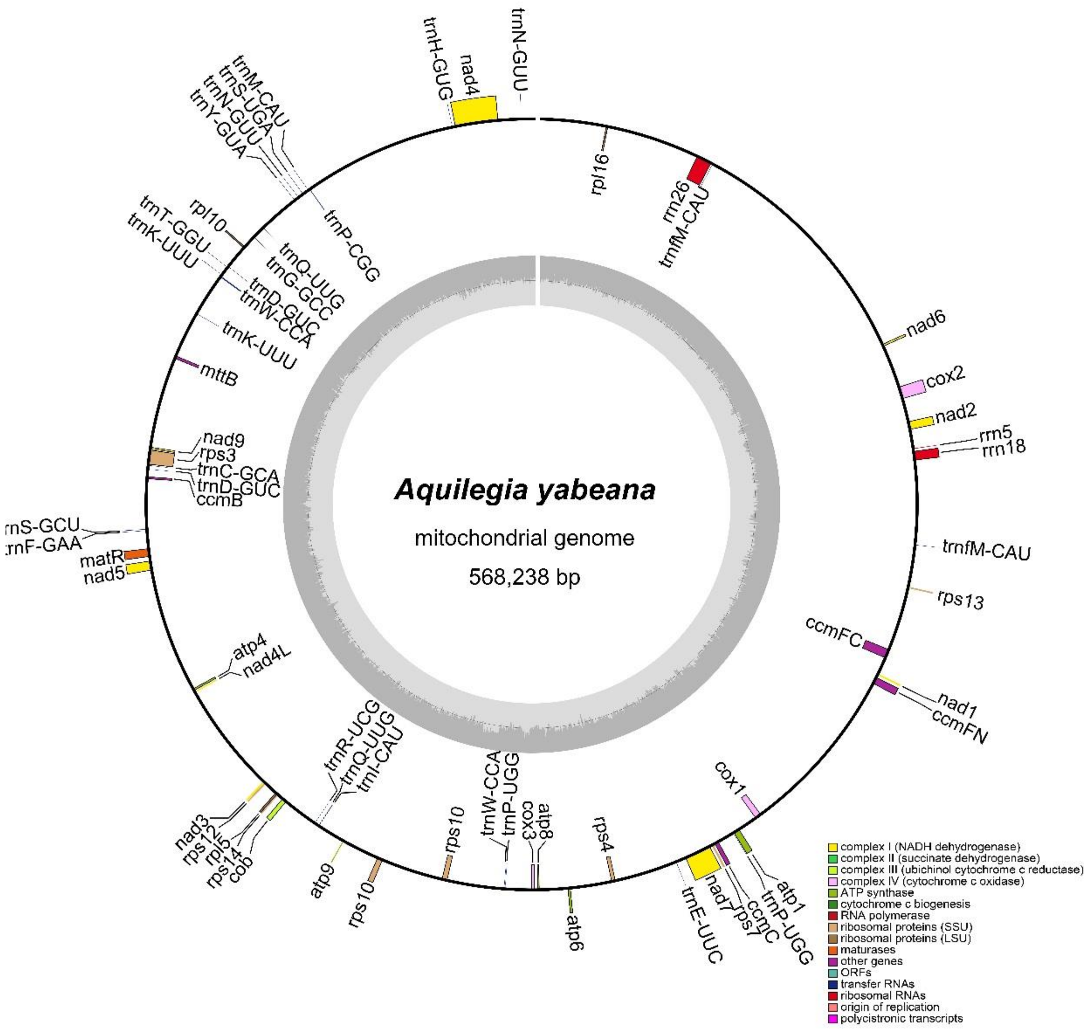

Figure S6. Schematic representation of the mitochondrial genomes of *A. yabeana*. Genes located on the outside of the circle are on the forward strand, whereas the genes inside circle are on the reverse strand. The genes are color-grouped according to functions.

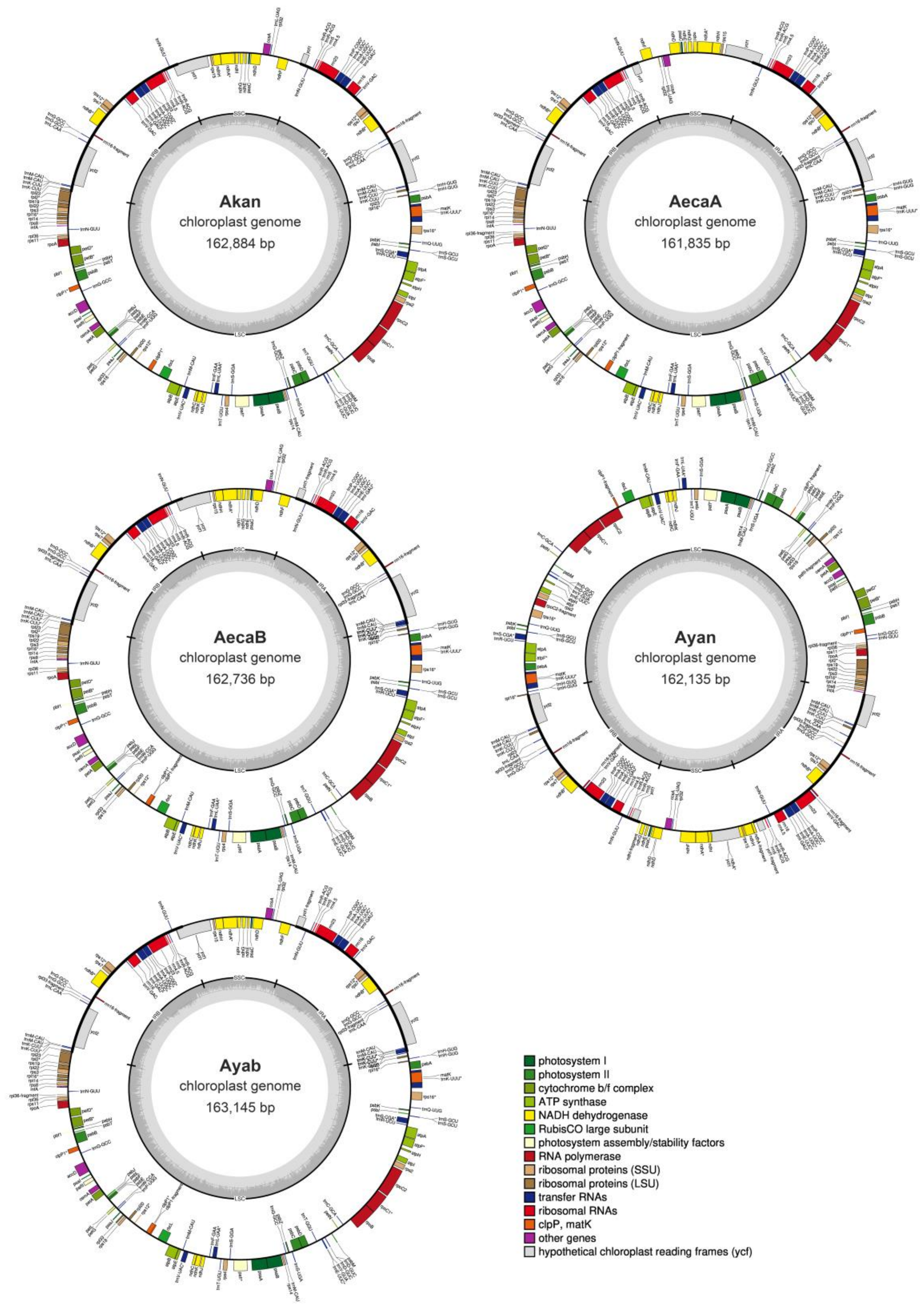

Figure S7 Schematic of the chloroplast genomes of *A. kansuensis* (Akan), *A. ecalcarata*_clade A (AecaA), *A. yanggi* (Ayan), *A. yabeana* (Ayab), and *A. ecalcarata*_clade B (AecaB).

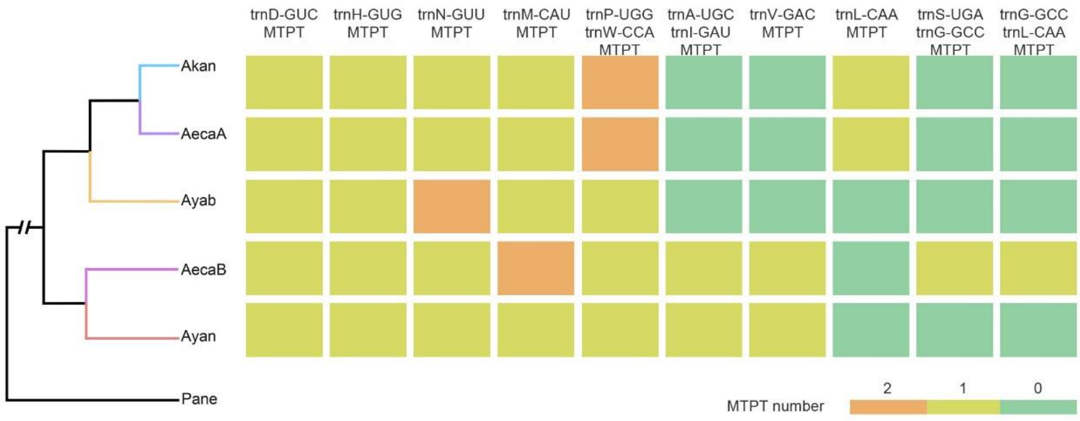

Figure S8 The heatmap of MTPT (containing tRNA genes) numbers of *A. kansuensis* (Akan), *A. ecalcarata*_clade A (AecaA), *A. yanggi* (Ayan), *A. yabeana* (Ayab), and *A. ecalcarata*_clade B (AecaB).

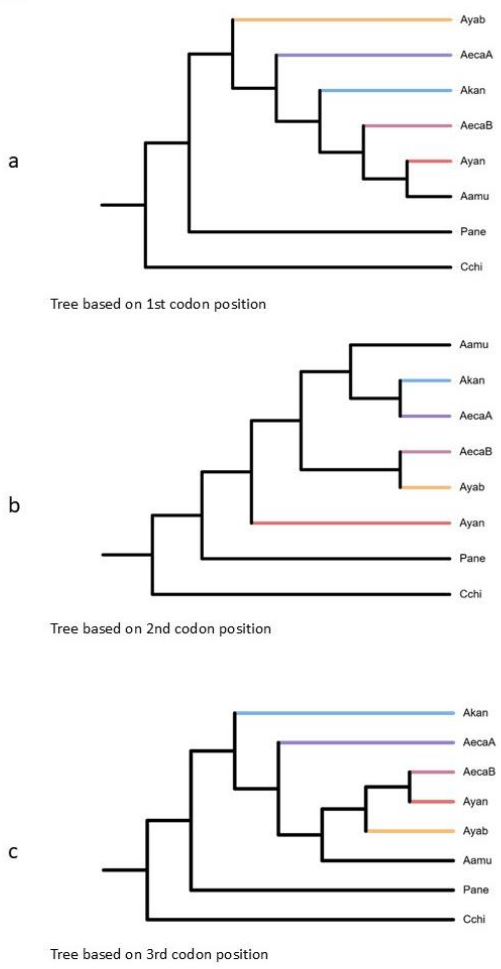

Figure S9 The codon-partitioned ML phylogenetic analysis result.
