# Supplementary material for: Assembly and Comparative Analysis of Five Complete Mitochondrial Genomes of the Aquilegia ecalcarata Complex
Source: Int J Mol Sci. 2026 Jul 24;27(15):6589. doi: 10.3390/ijms27156589 (PMC13464993; doi:10.3390/ijms27156589)
Supplement: Supplementary file 1 [file ijms-27-06589-s001.zip › Supplementary file S3_Alternative configurations and read-plot.pdf]

Akan

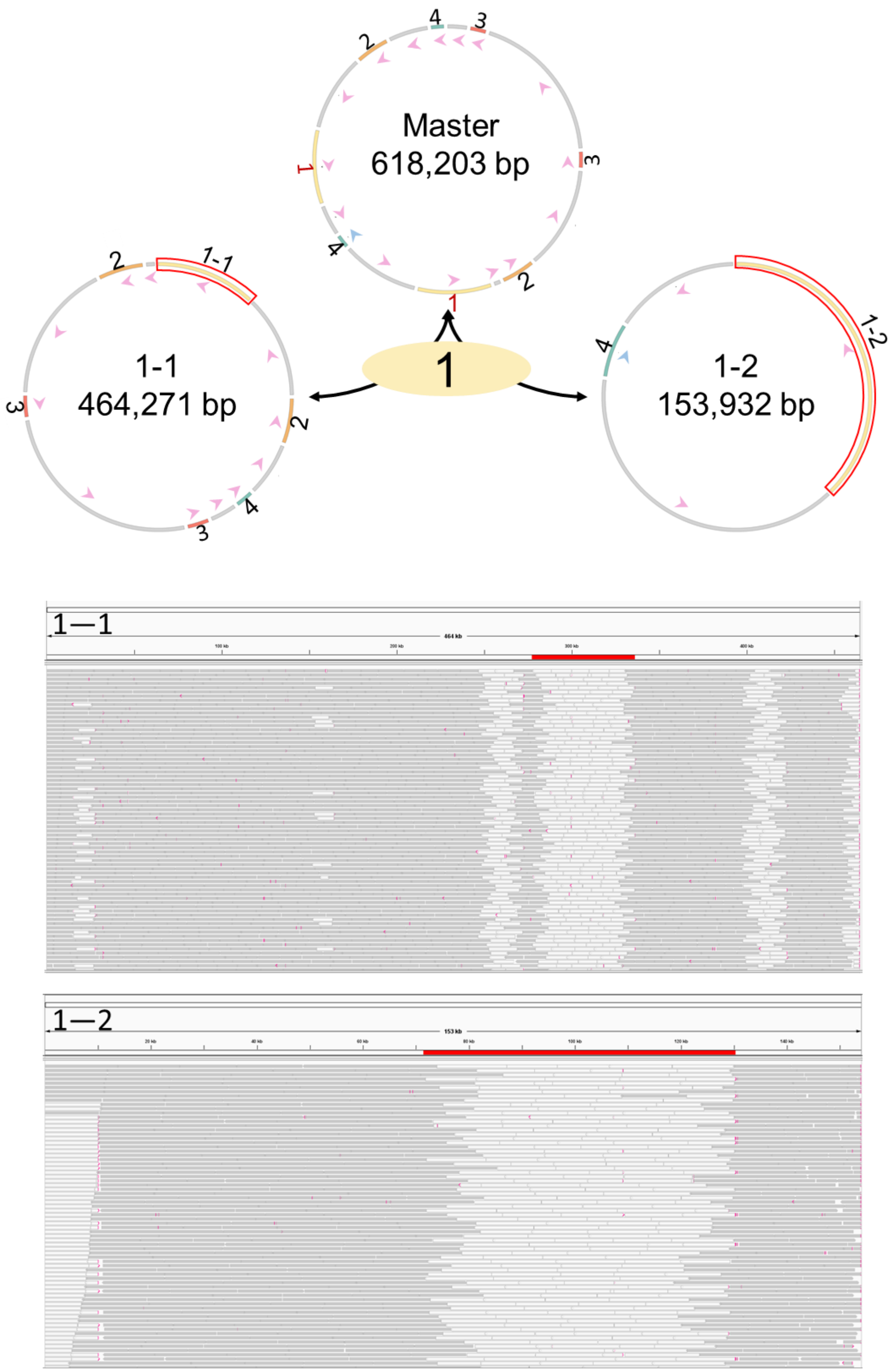

Different configurations mediated by the Akan repeat 1 pair, and corresponding read-plot.

Akan

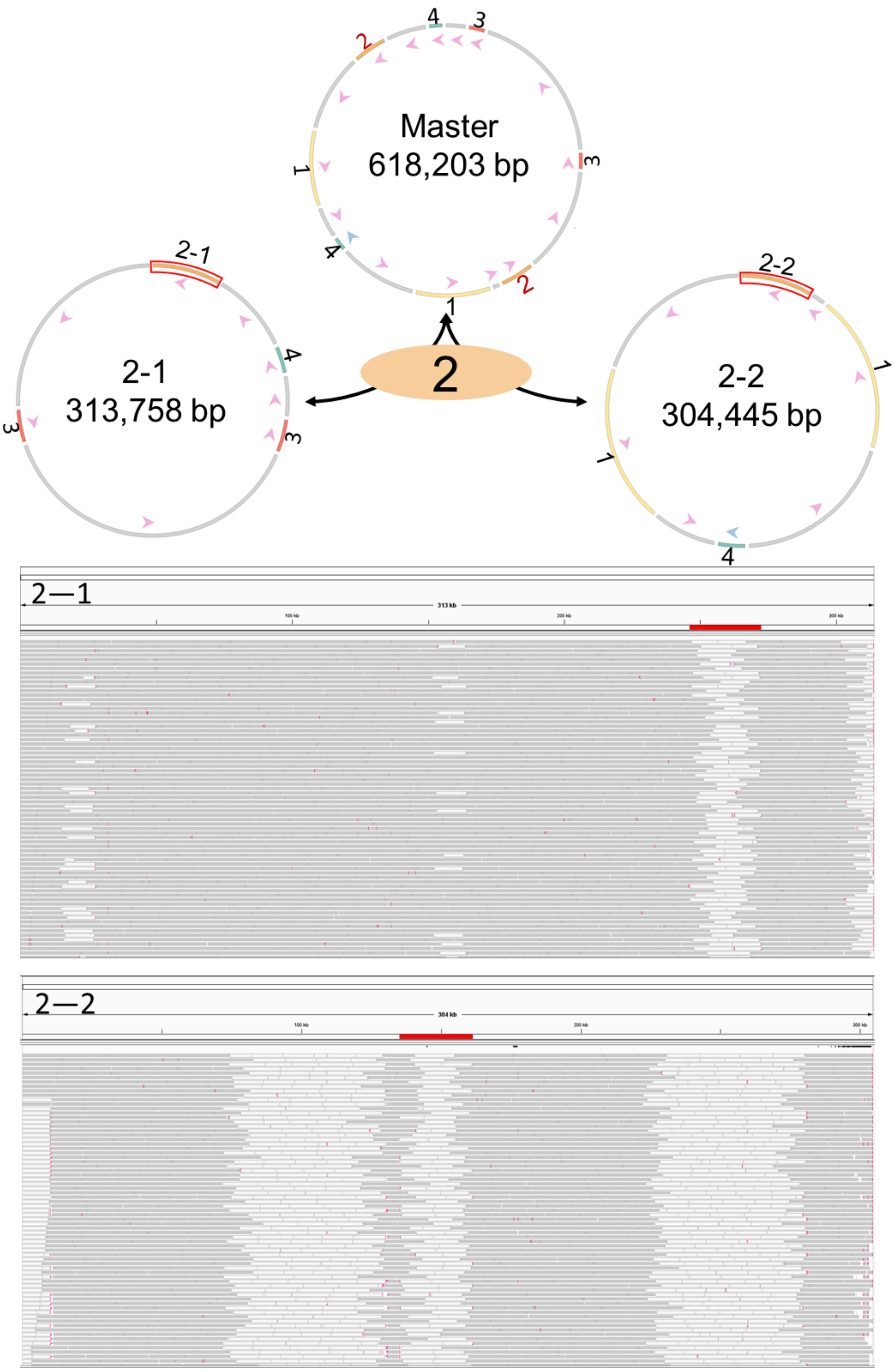

Different configurations mediated by the Akan repeat 2 pair, and corresponding read-plot.

Akan

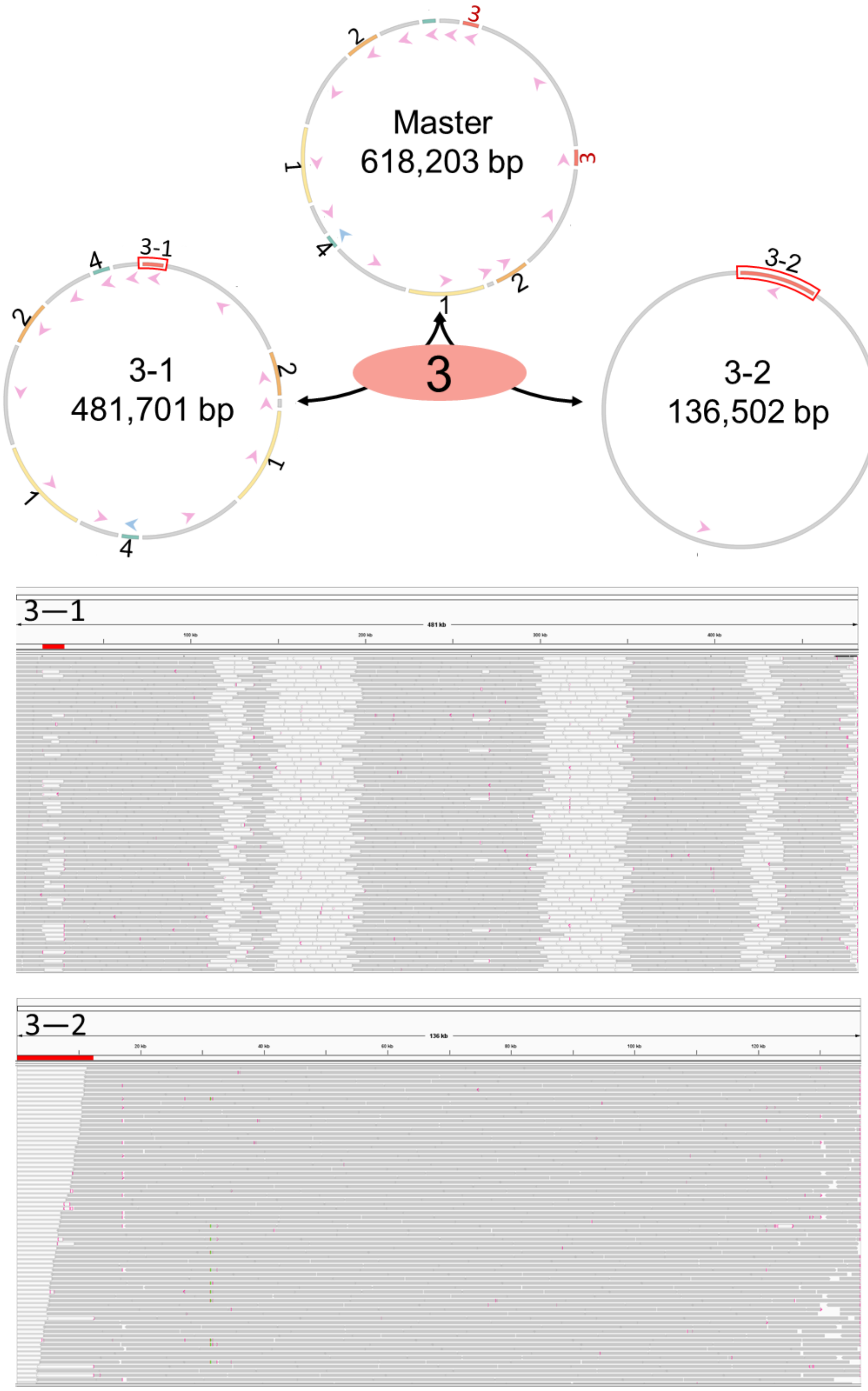

Different configurations mediated by the Akan repeat 3 pair, and corresponding read-plot.

Akan

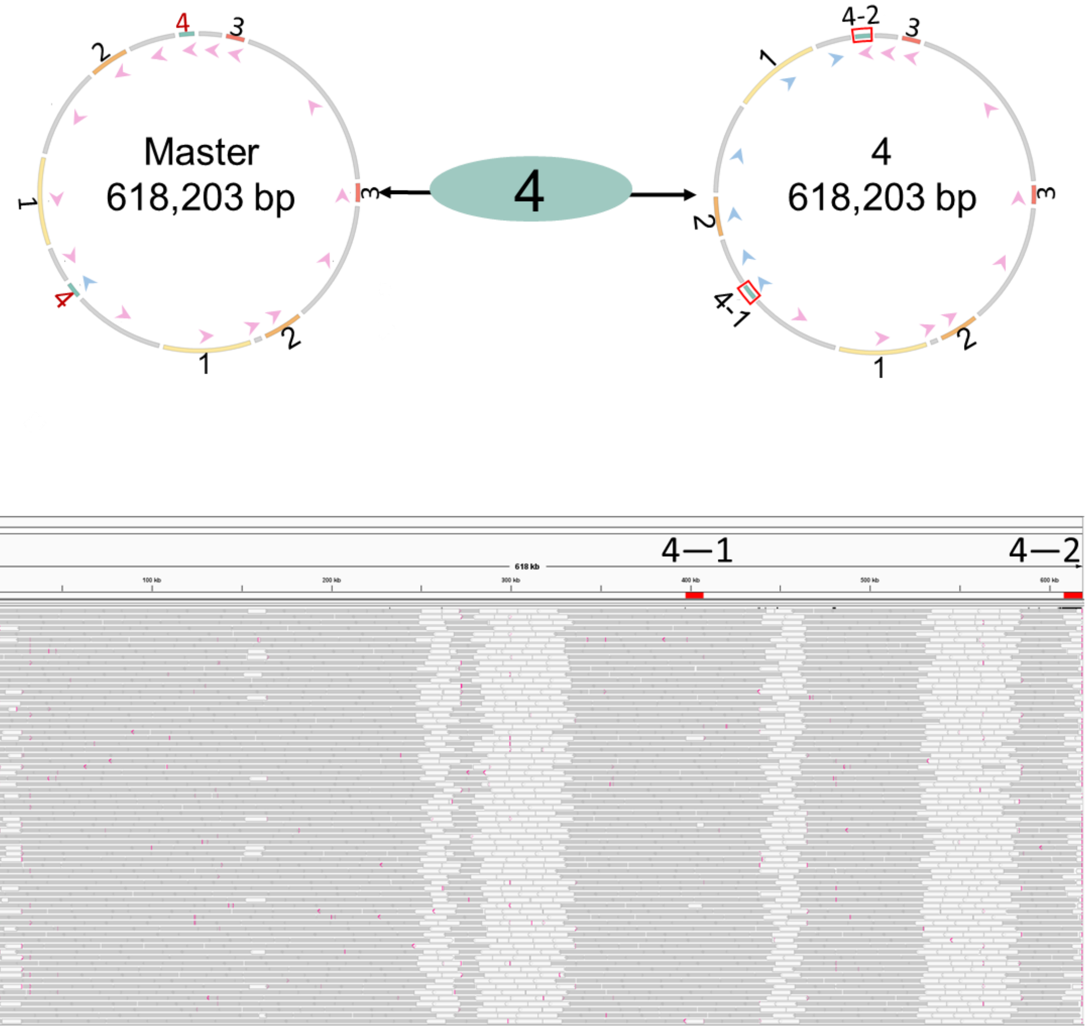

Different configurations mediated by the Akan repeat 4 pair, and corresponding read-plot.

AecaA

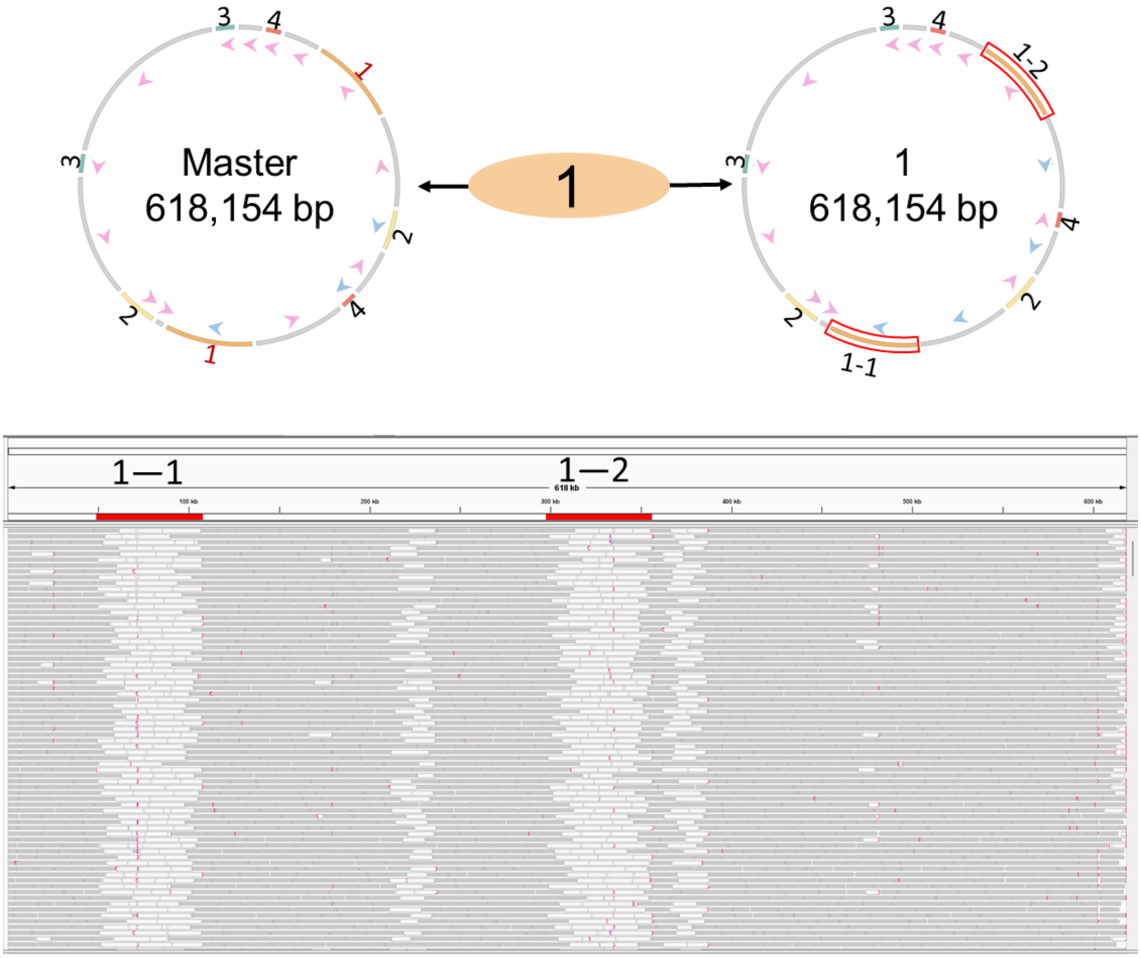

Different configurations mediated by the AecaA repeat 1 pair, and corresponding read-plot.

AecaA

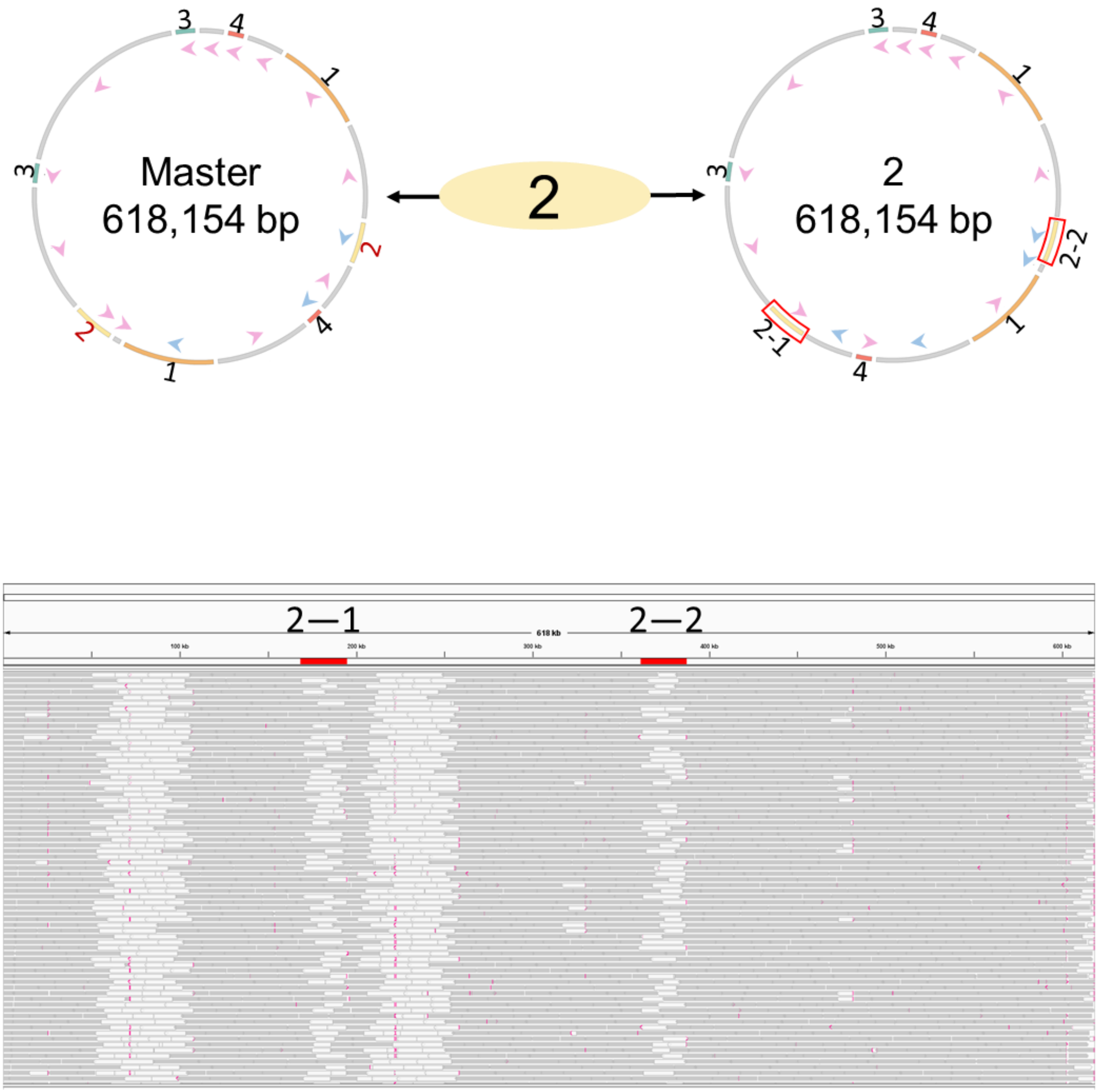

Different configurations mediated by the AecaA repeat 2 pair, and corresponding read-plot.

AecaA

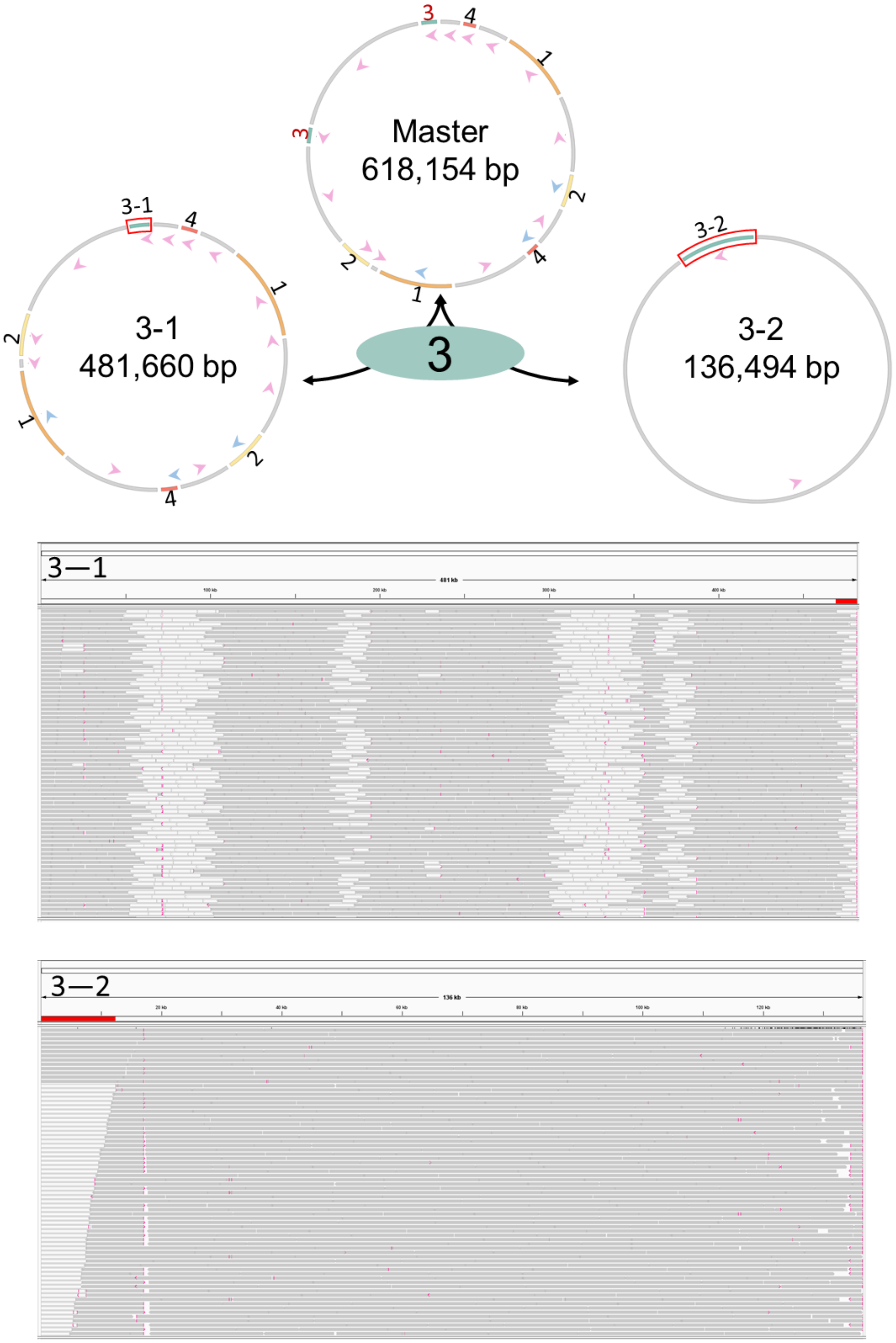

Different configurations mediated by the AecaA repeat 3 pair, and corresponding read-plot.

AecaA

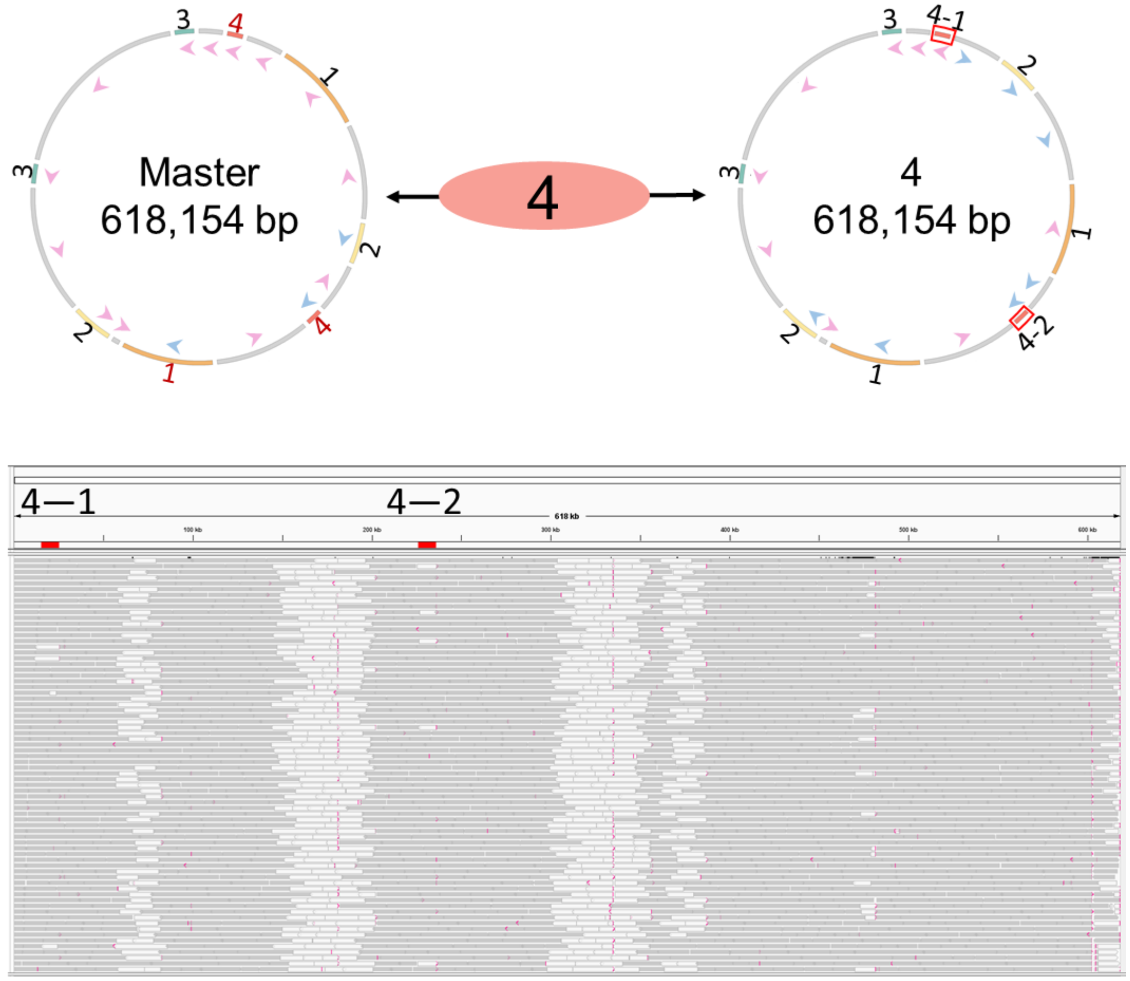

Different configurations mediated by the AecaA repeat 4 pair, and corresponding read-plot.

AecaB

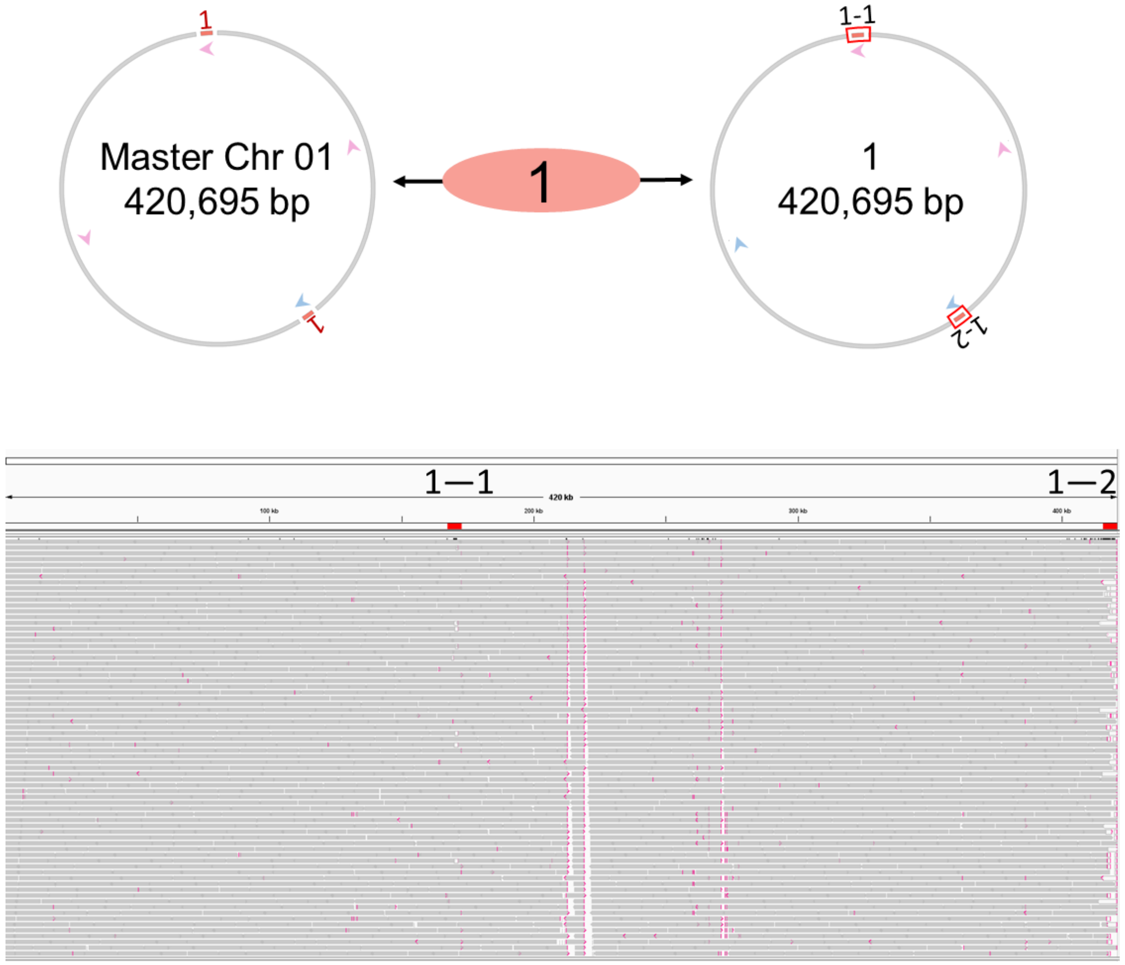

Different configurations mediated by the AecaB repeat 1 pair

AecaB

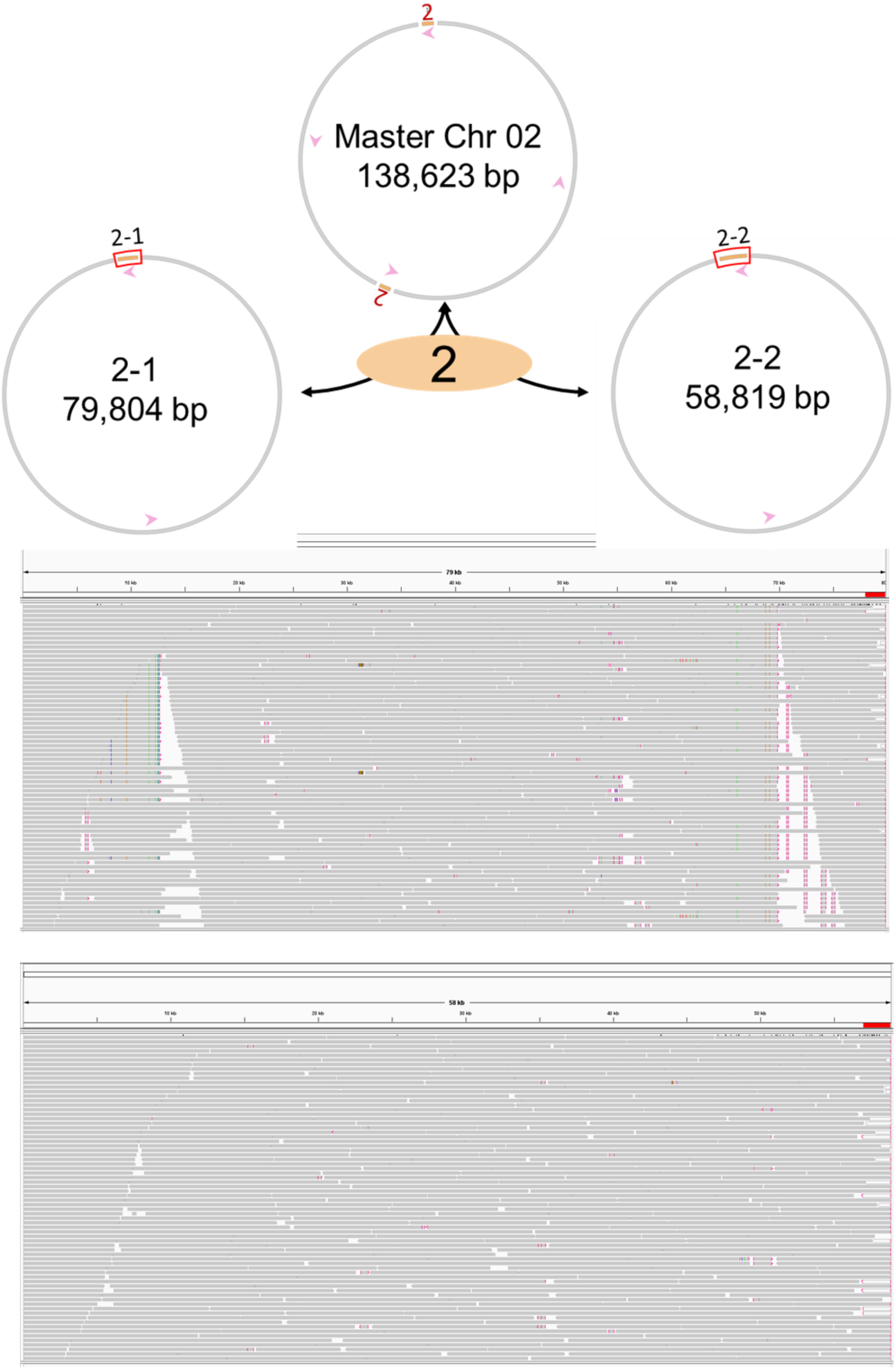

Different configurations mediated by the AecaB repeat 2 pair, and corresponding read-plot.

Ayan

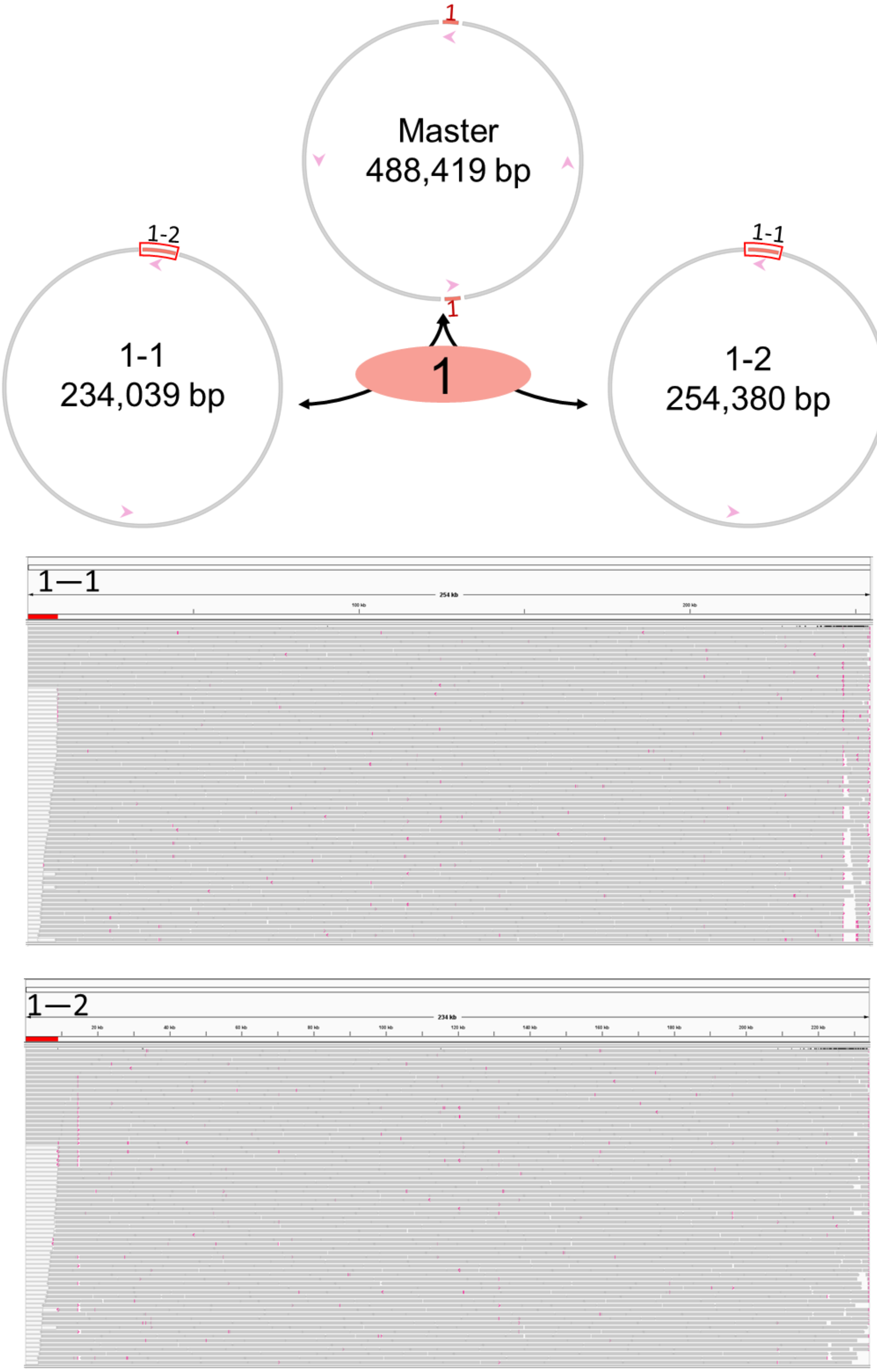

Different configurations mediated by the Ayan repeat 1 pair

Ayab

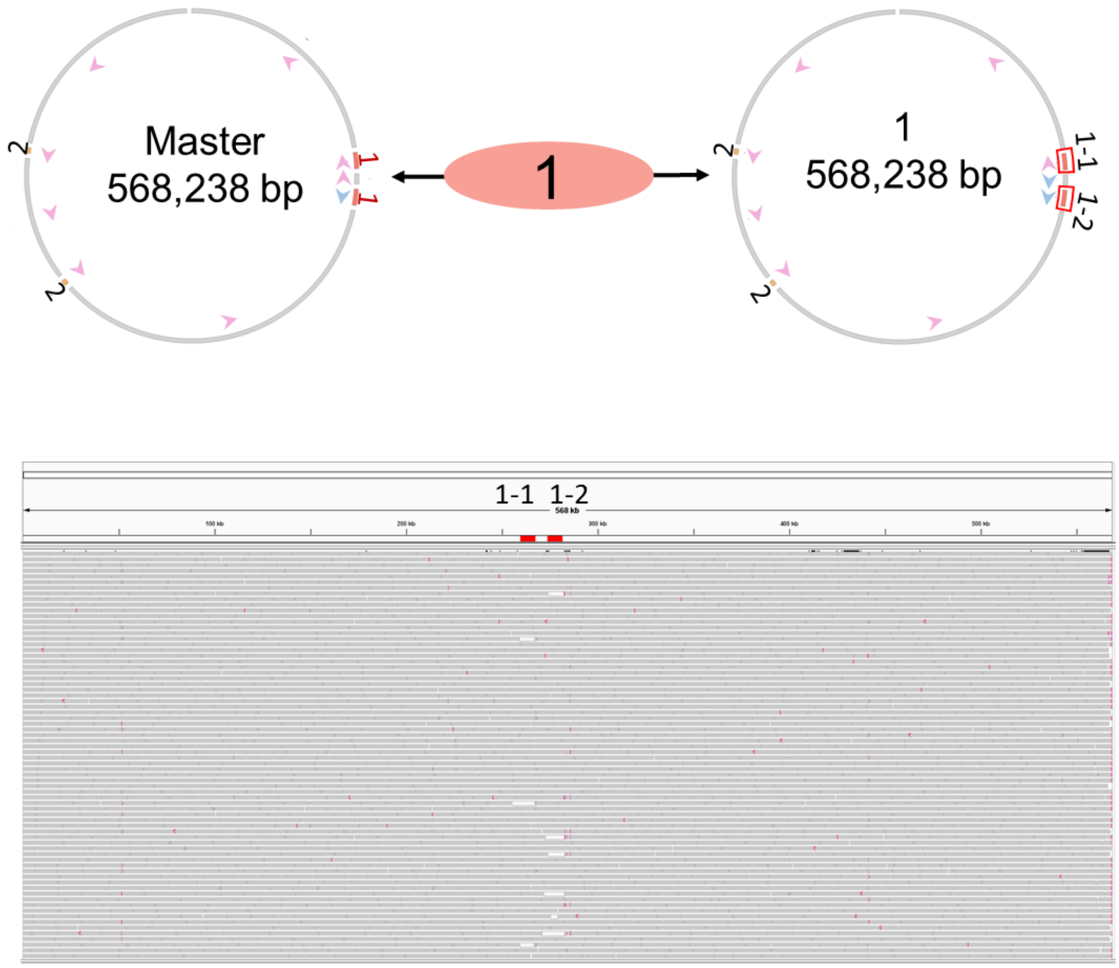

Different configurations mediated by the Ayab repeat 1 pair, and corresponding read-plot.

Ayab

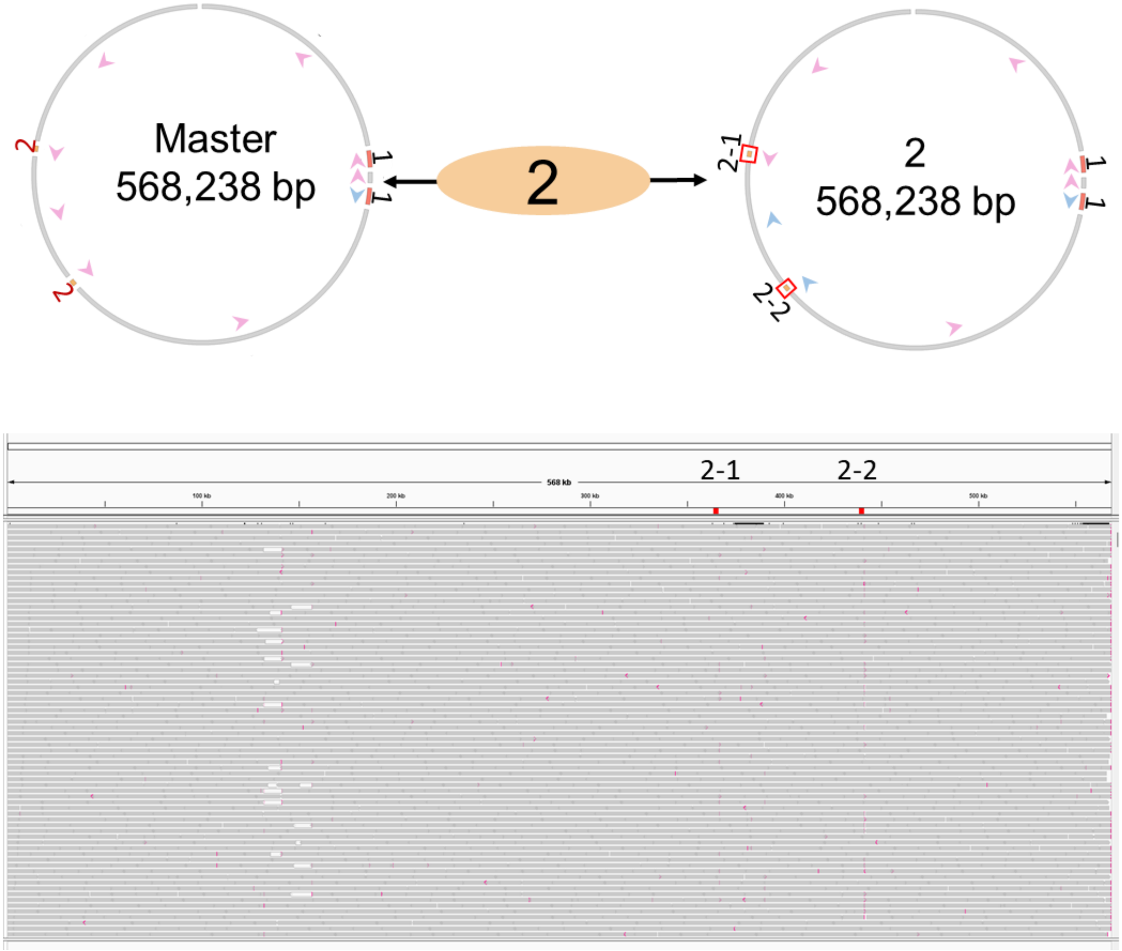

Different configurations mediated by the Ayab repeat 2 pair, and corresponding read-plot.
